# Functional characterization and developmental expression profiling of gibberellin signalling components in *Vitis vinifera*

**DOI:** 10.1093/jxb/eru504

**Published:** 2015-01-14

**Authors:** Atiako Kwame Acheampong, Jianhong Hu, Ariel Rotman, Chuanlin Zheng, Tamar Halaly, Yumiko Takebayashi, Yusuke Jikumaru, Yuji Kamiya, Amnon Lichter, Tai-Ping Sun, Etti Or

**Affiliations:** ^1^Institute of Plant Sciences, Department of Fruit Tree Sciences, Agricultural Research Organization, Volcani Centre, Bet Dagan 50250, Israel; ^2^Institute of Plant Sciences and Genetics in Agriculture, The Faculty of Agriculture, Food and Environment, The Hebrew University of Jerusalem, Rehovot 76100, Israel; ^3^Department of Biology, Duke University, Durham, North Carolina 27708, USA; ^4^RIKEN Plant Science Centre, Yokohama, Kanagawa 230-0045, Japan; ^5^Institute of Postharvest and Food Sciences, Department of Postharvest Science of Fresh Produce, Agricultural Research Organization, Volcani Centre, Bet Dagan 50250, Israel

**Keywords:** F-box proteins, GA receptors, gibberellins (GAs), gibberellin signalling, grapevine (*Vitis vinifera*), VvDELLA proteins.

## Abstract

Gibberellins (GAs) regulate numerous developmental processes in grapevine (*Vitis vinifera*) such as rachis elongation, fruit set, and fruitlet abscission. The ability of GA to promote berry enlargement has led to its indispensable use in the sternospermocarpic (‘seedless’) table grape industry worldwide. However, apart from VvGAI1 (VvDELLA1), which regulates internode elongation and fruitfulness, but not berry size of seeded cultivars, little was known about GA signalling in grapevine. We have identified and characterized two additional DELLAs (VvDELLA2 and VvDELLA3), two GA receptors (VvGID1a and VvGID1b), and two GA-specific F-box proteins (VvSLY1a and VvSLY1b), in cv. Thompson seedless. With the exception of VvDELLA3-VvGID1b, all VvDELLAs interacted with the VvGID1s in a GA-dependent manner in yeast two-hybrid assays. Additionally, expression of these grape genes in corresponding *Arabidopsis* mutants confirmed their functions *in planta*. Spatiotemporal analysis of VvDELLAs showed that both VvDELLA1 and VvDELLA2 are abundant in most tissues, except in developing fruit where VvDELLA2 is uniquely expressed at high levels, suggesting a key role in fruit development. Our results further suggest that differential organ responses to exogenous GA depend on the levels of VvDELLA proteins and endogenous bioactive GAs. Understanding this interaction will allow better manipulation of GA signalling in grapevine.

## Highlight

Characterization of grape GA signalling components and GA quantitation unveil the berry-size regulating DELLA, and show that differential organ responses stem from both VvDELLAs and endogenous GA levels.

## Introduction

Bioactive gibberellins (GA) are phytohormones involved in major physiological processes (Fleet and Sun, 2005; Yamaguchi, 2008). The most common bioactive GAs in higher plants are GA_1_, GA_4_, and GA_7_, a small subset of the more than 136 GAs identified (MacMillan, 2001). The richest sources of GAs in most plants are seeds (Dennis Jr. and Nitsch, 1966), inflorescences, and nodes (Kaufman *et al.*, 1976). In seeds, GA is synthesized in the embryo (Kaneko *et al.*, 2003), while the tapetum of anthers, stamens, flower receptacles, and rosette leaves constitute specific synthesis sites in flowering plants (Kaneko *et al.*, 2003; Hu *et al.*, 2008). The metabolic processes regulating GA biosynthesis and/or deactivation in these organs are tightly controlled by its concentration, as well as developmental, hormonal, and environmental cues (Yamaguchi, 2008). Generally, GA levels are elevated in reduced GA-response mutant backgrounds, and vice versa (Thomas *et al.*, 1999; Ueguchi-Tanaka *et al.*, 2005; Griffiths *et al.*, 2006).

The involvement of GAs in grapevine (Vitis vinifera) berry development and size determination was first described by Coombe (1960). In the berries of seeded cultivars, GAs are produced by the seeds (Lavee, 1960). Application of GA to seeded grape cultivars prior to anthesis increases fruit set and induces parthenocarpy (Chundawat *et al.*, 1971); causes lignification and contortion of the rachis (Agüero *et al.*, 2000); and induces berry enlargement in some cultivars (Nijjar and Bhatia, 1969), while reducing berry size in others (Chundawat *et al.*, 1971).

In stenospermocarpic cultivars, which represent the majority of the ‘seedless’ commercial cultivars, fertilization is followed by endosperm abortion, which occurs early during fruit development and leads to cessation of seed development. In these cultivars, seeds serve as the primary source of GA only prior to abortion (Conde *et al.*, 2007). Thus, the berries of these cultivars are usually small with relatively low levels of GA (Iwahori *et al.*, 1967). To enhance berry size and viticulturally important traits such as rachis elongation and berry abscission, seedless cultivars have been treated with GA since the late 1950s (Weaver, 1958). However, GA application at other developmental stages can have negative effects on reproductive development in grapes: when applied at anthesis GAs may induce formation of shot-berry, and enhance berry abscission (Mosesian and Nelson, 1968). In addition, early application of GA to shoots may reduce fruitfulness due to the development of uncommitted primordia to tendrils (Srinivasan and Mullins, 1981).

Seedless cultivars show a wide range of sensitivity to GA treatment (Wolf and Loubser, 1992). Parallel to these varietal differences, differential GA sensitivity has also been reported in tissues/organs within the cultivar (Mullins *et al.*, 1992; Agüero *et al.*, 2000), although the basis for these differences remains unknown. To understand the underlying mechanism of GA response in grapevine in general, and in the berry in particular, it is imperative to elucidate the grapevine GA signalling components. Recent studies showed that GA receptors and the early GA signalling pathway are highly conserved in higher plants (Hirano *et al.*, 2008; Sun, 2011). The GA signal is perceived by its nuclear receptor GA INSENSITIVE DWARF1 (GID1). GA activates its signalling pathway by promoting interaction between GID1 and DELLA, a GA signalling repressor. Binding of GA-GID1 to DELLA induces recognition of DELLA for ubiquitination by a specific F-box protein (SLY1 in *Arabidopsis* and GID2 in rice), a subunit of the ubiquitin E3 ligase SCF complex. Once polyubiquitinated, DELLA is degraded rapidly by the 26S proteasome. Thus far, the only characterized GA signalling component in grapevine has been VvGAI1 (Boss and Thomas, 2002), which is a DELLA protein that represses internodes and rachis elongation, but has no effect on berry size. This implies that other component(s) of the GA signalling cascade may regulate grape berry size.

In this study, we isolated and functionally characterized all the grapevine homologues of DELLA, GID1, and SLY1. We also carried out spatiotemporal analysis of *VvDELLA*, *VvGID1*, and *VvSLY1* transcripts, and of VvDELLA proteins, and investigated their regulation in response to GA_3_ and the GA biosynthesis inhibitor [paclobutrazol (PAC)] treatments. One of the major findings of this study is that the response of an organ to GA_3_ application depends on the total amount of VvDELLA proteins and bioactive GAs in the organ.

## Materials and methods

### Field experiment and sampling

The experiments were conducted in the 2010 and 2011 growing seasons, on 10-year-old Thompson seedless vines (*Vitis vinifera* L.) growing in a vineyard in Moshav Lachish, Israel (N31°33′33; E34°51′26). The planting system was composed of Y-shaped trellis with planting distances of 3×1 m on Richter 110 (*V. berlandieri × V. rupestris*) rootstock. Standard cultural practices were applied in the vineyard. All experiments were replicated three times using eight-vine plots arranged in a randomized complete-block design.

#### Treatment with GA_3_ and PAC

Groups of 25 uniform 15cm shoots and 25 inflorescences [E-L 15, on the Modified Eichhorn and Lorenz system (Coombe, 1995)] were selected on vines of similar vigour for internode and rachis experiments, respectively. Inflorescences at E-L 17 and clusters at E-L 27 were selected for carpel and berry experiments, respectively. Organs received a single Triton X-100 (0.025%)-formulated GA_3_ (Pro-Gibb 4%; Abbott Laboratories, Chicago, USA) or 0.8mM PAC (CULTAR 25 SC, Syngenta AG, Basel, Switzerland) application. Rachises and internodes were treated with 121 µM GA_3_, while carpels and berries were treated with 90 µM GA_3_. To allow effective inhibition of GA biosynthesis, PAC was applied 4 days before GA_3_ and control treatments. PAC-GA treatment was included as well, where samples received GA_3_ application 4 days after PAC treatment. This combined treatment was carried out to verify that growth inhibition by PAC is mainly due to decreased GA biosynthesis, and thus can be reversed if exogenous GA is applied. Triton X-100-treated organs served as controls. All organs were treated either by dipping or spraying to the point of run-off.

##### Morphological response of organs to GA_3_ and PAC

Pre-treatment lengths of rachises, and weights of berries, were recorded. Increments in the lengths of rachises and the newest internodes arising after treatment were monitored at 5-day intervals, while berry weights were assessed at 10-day intervals for 30 days.

##### Sampling for GA response/signalling analyses

Organs and tissues were sampled 6h after GA treatments. All samplings were done before 14:00 to minimize circadian effects on gene expression. Sampled organs and tissues were immediately frozen in liquid nitrogen in the vineyard, and stored at –80°C prior to analyses.

##### Sampling for temporal and spatial analyses, and GA quantitation

Sampling of young internodes was carried out from the most distal internodes from the base of young shoots at E-L 15, while young rachises were sampled from inflorescences at E-L 15. Tissues and organs at similar developmental stages were marked and sampled at véraison and defined as mature internodes and rachis. Young leaves and tendrils were defined as those borne on the first and second nodes (from the shoot tip), while mature leaves and tendrils were sampled from the 12th node. Carpels were sampled at E-L 17, while berries were sampled at E-L 27, and subsequently at 10 and 30 days after the first sampling, herein referred to as 0, 10 and 30 days after fruit set (DAF), respectively. Roots were obtained from single node cuttings immersed in water for about 21 days. Samples were collected before 09:00 to minimize circadian effects on gene expression.

### Phylogenetic analysis of genes associated with GA signalling

Multiple alignment of protein sequences of the *Arabidopsis*, rice and *V. vinifera* families were generated using the CLUSTAL W alignment algorithm (Thompson *et al.*, 1994) using AlignX of the Vector NTI suite (Lu and Moriyama, 2004). Phylogenetic unrooted trees were constructed using the neighbour-joining (NJ) method in the web-based Phylogeny.fr software (http://phylogeny.lirmm.fr/phylo_cgi/index.cgi; last accessed: 22 December 2014) (Dereeper *et al.*, 2008).

### Gene cloning and plasmid construction

Total RNA was extracted using the CTAB protocol as previously described (Acheampong *et al.*, 2010). First-strand cDNA was synthesized using Moloney Murine Leukemia Virus Reverse Transcriptase (M-MLV RT) (Promega Corporation, Madison, WI, USA) according to the manufacturer’s instructions. Full-length ORFs of all genes were PCR-amplified from cDNA from different organ primer sets listed in Supplementary Table S1. PCR fragments were amplified with primers having the recommended GATEWAY overhangs, cloned into pENTR/D-TOPO or pENTR/SD/D-TOPO vectors (Invitrogen, Carlsbad, CA, USA), and subsequently cloned into the GATEWAY-based, 35S-driven pK7WG2.0 overexpression vector (Karimi *et al.*, 2002) or pET-DEST42 protein expression vector (Invitrogen). The overexpression constructs were introduced into *Agrobacterium tumefaciens* strain GV3101 by electroporation. To ensure the specificity of the anti-VvDELLA polyclonal antibodies, sequences encoding differential domains were used (VvDELLA1, T101-V205; VvDELLA2, P113-Q226; VvDELLA3, V55-D151). These recombinant His-tagged constructs were expressed in BL21-CodonPlus (DE3) RIPL strains (Strategene, Santa Clara, CA, USA). The full lengths of the VvDELLA genes were also PCR amplified, cloned into the Entry and Destination vectors, and transformed into BL21-CodonPlus (DE3) RIPL cells.

For the yeast two-hybrid (Y2H) assay, pLexA-NLS and pACTII were used as bait and prey expression vectors, respectively, as described previously (Dill *et al.*, 2004). VvGID1 and VvSLY1 proteins were expressed in pLexA-NLS as fusions with LexA DNA binding domain (DNA-BD), while VvDELLA proteins were expressed in pACTII as fusions with the GAL4 activation domain (AD). Primers for Y2H cloning, with appropriate restriction sites are listed in Supplementary Table S2.

*Arabidopsis* transformation of grape genesUsing the *A. tumefaciens*-mediated transformation method, 35S promoter-driven grape cDNA constructs were transformed into corresponding *Arabidopsis* mutants for complementation tests. *35S:VvGID1* was transformed into *gid1a-2 gid1c-2* mutants (Griffiths *et al.*, 2006). *35S:VvSLY1* was transformed into *sly1-10 +/–* plants due to severe infertility of the *sly1-10* homozygote line (Steber *et al.*, 1998). Later the *sly1-10* homozygote allele was identified in T2 generations of *VvSLY1* transformants. *35S:VvDELLA* was transformed into the *ga1-3 rga-24* mutant (Silverstone *et al.*, 1998). Isolation of homozygous transgenic lines containing single insertion sites was done as described previously (Hu *et al.*, 2008).

### Y2H assays

DNA-BD and AD fusion construct co-transformation into *Saccharomyces cerevisiae* strain L40, growth testing with 3-AT, and β-galactosidase liquid assaying were carried out as described previously (Dill *et al.*, 2004).

### Quantitative real-time PCR analyses

The transcript levels of *VvGID1*s, *VvDELLA*s, and *VvSLY1*s were measured by quantitative real-time PCR (qRT-PCR) using Takara SYBR-green Premix Ex Taq (Thermo Fischer Scientific, Waltham, MA, USA) on the Rotor Gene 6000 Real-time PCR machine (QIAGEN, Hilden, Germany). The previously characterized *VvGAPDH* (Reid *et al.*, 2006) was used as a normalizer; its expression in grapevine organs is not GA regulated (Giacomelli *et al.*, 2013), and its orthologue in *Arabidopsis* was found to be unaffected by GA (Dill *et al.*, 2004). The SYBR-Green reaction mixture consisted of 12 µl of 0.5 µM forward and reverse primers (Supplementary Table S1), 6 µl of SYBR-Green, and 3 µl of first-strand cDNA. PCR reactions were performed using the following parameters: 15min at 95°C, and 40 cycles of 15 s at 95°C, 20 s at 60°C, and 20 s at 72°C. Each sample was analysed six times, comprising three biological repeats with two technical repeats each. To ensure accurate quantitation of transcripts, we selected and used primers of comparable efficiencies. We also synthesized a plasmid containing the amplicons of all qRT-PCR products of the genes (cloned into the PUC19 vector), linearized it by digesting with BamHI restriction enzyme (NEB, Ipswich, MA, USA), and used it as a template for gene-specific calibration curves, as briefly described below. From an initial concentration of 23 pmol l^–1^ (42 816 420 copies), 10-fold serial dilutions were made and used as templates. Amplicons were confirmed by agarose gel electrophoresis and sequencing.

### Antibody production

Cloning of constructs for protein induction was as described above. Proteins were expressed from constructs encoding differential regions of each VvDELLA protein using 0.5mM IPTG and 200 µg ml^–1^ rifampicin as previously described (Kuderova *et al.*, 1999). The His-tagged recombinant proteins were subsequently purified using a QIAGEN protein purification kit as detailed in the protocol (QIAGEN). The expressed proteins were sequenced by mass spectrometer to ensure sequence integrity, and used as antigens to produce affinity-purified rabbit anti-VvDELLA polyclonal antibodies. Production and purification of polyclonal antibodies were contracted to GenScript USA Inc (Piscataway, NJ, USA). Recombinant full-length VvDELLA proteins, used as sizing standards to locate endogenous VvDELLA proteins, were also expressed and purified as described above, and quantified using BSA standards.

### Protein extraction and immunoblot analyses of VvDELLA proteins

Total plant protein was extracted from organs as previously described (Wang *et al.*, 2006) with slight modifications. Samples were first homogenized in liquid nitrogen in the presence of polyvinyl polypyrrolidone (PVPP). The protein pellets obtained were dissolved in SDS-PAGE sample buffer containing 0.15M Tris (pH 6.8), 1.2% SDS, 30% glycerol, 2.14M β-mercaptoethanol (Sigma Aldrich, St Louis, MO, USA). Extracted proteins were quantified by band intensities confirmed by fractionating on 10% SDS-PAGE gel, and staining with Coomassie protein staining buffer (0.1% Coomassie Brilliant Blue R-250, 50% methanol and 10% glacial acetic acid). Equal amounts of proteins were separated by 10% SDS-PAGE and transferred to PROTEAN nitrocellulose transfer membrane (Whatman GmbH, Dassel, Germany) using the Mini-Protean Transfer system (Bio-Rad Laboratories, Hercules, CA, USA). Detection of protein was as described by Yamamoto *et al.* (2010) with slight modifications. Blotted membranes were blocked in 3% protein solution (3% milk powder, 50mM Tris-HCl pH 7.5, 150mM NaCl, 0.05% Tween 20) for 1h, and the primary antibody titres were 1:2000, 1:1000, and 1:5000 for VvDELLA1, VvDELLA2, and VvDELLA3, respectively. Purified full-length recombinant proteins of VvDELLA1 (3.75ng), VvDELLA2 (3.75ng), and VvDELLA3 (37.5ng) were used to identify and quantify endogenous VvDELLA proteins for each blot. Chemi-luminescence images of blots were captured by MF-ChemiBIS 2.0 (DNR Bio-Imaging Systems Ltd, Jerusalem, Israel).

### Quantitation of endogenous gibberellins

Triplicate samples (0.5g) of freeze-dried organs/tissues were weighed, homogenized in liquid nitrogen, and extracted with 80% methanol containing 1% acetic acid and ^2^H-labelled GAs as internal standards (Supplementary Table S3), for 1h at 4°C. Samples were centrifuged at 3000*g* for 10min, and filtered through LRC-2 Frits Bond Elut Reservoir (Varian Inc, Palo Alto, CA, USA) to remove residual plant materials. The solvent (80% methanol, 1% acetic acid) extraction was repeated for 10min, and samples centrifuged and filtered as before. The two extracts were combined and evaporated to dryness at 35°C using Savant SpeedVac Concentrators (Thermo Fischer Scientific). Dried samples were dissolved in 1ml of 80% acetonitrile, 1% acetic acid. The acetonitrile was removed by evaporation *in vacuo*.

Endogenous bioactive GAs, their precursors, and deactivation products were measured as previously described (Plackett *et al.*, 2012). The prominent ions were analysed by a liquid chromatography tandem mass spectrometry system consisting of a triple quadrupole mass spectrometer (Agilent 6410; Agilent Technologies, Santa Clara, CA, USA) and an Ultra High Performance Liquid Chromatography (UHPLC) system equipped with an octylphenyl column (ZORBAX XDB-Phenyl, 2×50mm, 1.8 µm; Agilent Technologies). The endogenous GA contents were calculated from the peak area ratios of the endogenous GA to internal standards spiked during the extraction process.

### Statistical analyses

Unless otherwise stated, all experiments were designed with a completely randomized distribution. Data are presented as the mean ± standard error (SE). Statistical significance for data from field experiments was determined by one-way analysis of variance (ANOVA) followed by Tukey multiple comparison tests (JMP 7.01 software, SAS Institute, Cary, NC, USA), and significance values set at α = 0.05 as indicated in the text and figure legends.

### Accession numbers

Sequence data can be found in the NCBI GenBank data libraries under accession numbers: VvGID1a (AM468374), VvGID1b (AM479851), VvDELLA1 (AM459432.1), VvDELLA2 (AM470304.2), VvDELLA3 (AM484828.1), VvSLY1a (AM445694.2), VvSLY1b (AM450967), OsGID1 (Os05g0407500), AtGID1a (At3g05120), AtGID1b (At3g63010), AtGID1c (At5g27320), AtGAI (At1g14920), AtRGA (At2g01570), AtRGL1 (At1g66350), AtRGL2 (At3g03450), AtRGL3 (At5g17490), SLR1 (Os03g0707600), AtSLY1 (At4g24210), and OsGID2 (Os02g0580300). Descriptions of the grapevine genes are listed in Supplementary Table S4.

## Results

Grapevine GA signalling genesBioinformatic analyses were performed on the *V. vinifera* cDNA database (http://compbio.dfci.harvard.edu/tgi/; last accessed: 20 July 2012) and two independent genome sequence projects (Jaillon *et al.*, 2007; Velasco *et al.*, 2007), using sequence information of the characterized orthologues in rice and *Arabidopsis* from NCBI (http://www.ncbi.nlm.nih.gov/; last accessed: 23 December 2014). These databases revealed two putative homologues encoding GA receptor genes (VvGID1a and VvGID1b), two F-box proteins (VvSLY1a and VvSLY1b), and two putative DELLA genes (VvDELLA2 and VvDELLA3), in addition to the previously characterized VvGAI1 (Boss and Thomas, 2002), herein referred to as VvDELLA1 (Supplementary Table S4).

### VvGID1 genes and proteins

**Fig. 1.**
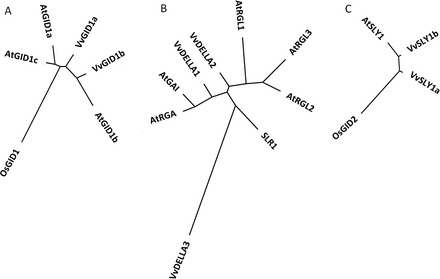
Neighbour-joining tree from the amino acid sequence alignment of GA signalling components isolated from *V. vinifera*. The tree was created by web-based Phylogeny.fr software (http://www.phylogeny.fr/version2_cgi/simple_phylogeny.cgi?tab_index=1). Sequence alignments were performed using the CLUSTALW2 program of MUltiple Sequence Comparison by Log-Expectation (MUSCLE), and phylogeny by PhyML programs. (A) A phylogentic tree containing VvGID1 paralogues (VvGID1a, VvGID1b), and orthologues from *Arabidopsis* (AtGID1a, AtGID1b, AtGID1c) and rice (OsGID1) (B) A phylogenetic tree containing VvDELLA paralogues (VvDELLA1, VvDELLA2, and VvDELLA3), and orthologues from *Arabidopsis* (AtGAI, AtRGA, AtRGL1, AtRGL2, AtRGL3), and the solitary rice orthologue, SLR1. (C) A phylogentic tree containing VvSLY1 paralogues (VvSLY1a and VvSLY1b), and orthologues from *Arabidopsis* (AtSLY1) and rice (OsGID2).

A BLAST search showed VvGID1a and VvGID1b protein sequences with high sequence homology to *Arabidopsis* AtGID1 (Griffiths *et al.*, 2006; Nakajima *et al.*, 2006) and rice OsGID1 (Ueguchi-Tanaka *et al.*, 2005). While each *VvGID1* paralogue encodes a 344 amino acid protein, and has a single intron 38 nucleotides downstream of the start codon, there are differences in their genomic architecture. *VvGID1a* is located on chromosome 14, and its intron size is 900bp meaning it is encoded by a 1935bp sequence, while *VvGID1b* is located on chromosome 7, and is encoded by a 1817bp sequence containing a 782bp intron. The amino acid sequences of the two VvGID1 paralogues are 79.1% identical, and share sequence homology with AtGID1a-1c and OsGID1 in conserved GA-binding residues (Suppementary Figure S1A). Like most angiosperms, VvGID1a is clustered with the ‘GID1ac’ group, whereas VvGID1b is clustered with the ‘GID1b’ group (Voegele *et al.*, 2011) on the phylogenetic tree (Fig. 1A). Similar to OsGID1 (Ueguchi-Tanaka *et al.*, 2005) and AtGID1s (Nakajima *et al.*, 2006), both VvGID1s have the conserved HGG and GxSxG motifs characteristic of hormone-sensitive lipases (HSLs), and also possess two (Ser-190 and Glu-288) of the three amino acids (Ser, Glu, His) that constitute the so-called catalytic triad of the HSL family. Most other functionally important residues previously described for GID1 (Ueguchi-Tanaka *et al.*, 2005; Nakajima *et al.*, 2006; Murase *et al.*, 2008) are present in both paralogus. A difference should be noted in position 128, where Thr in VvGID1a is replaced by Tyr in VvGID1b (Supplementary Figure S1A).

### VvDELLA genes and proteins

Similar to *Arabidopsis* RGA and rice SLR1, there are no introns in the genes encoding the 64.8kDa, 66.1kDa, and 58.6kDa proteins of VvDELLA1, VvDELLA2, and VvDELLA3, respectively. VvDELLA2 and VvDELLA3 share sequence similarity with the already characterized VvDELLA1 (Boss and Thomas, 2002), and the orthologues in *Arabidopsis* (RGA, GAI, RGL1, RGL2, and RGL3) and rice (SLR1) (Fig. 1B; Supplementary Figure S1B). Whereas the deduced amino acid sequences of VvDELLA1 and VvDELLA2 were 66% identical, both paralogues were ~36% identical to VvDELLA3. All three VvDELLAs possess the distinct triad of motifs required for GID1–DELLA interactions (Murase *et al.*, 2008): DeLLaΦLxYxV, MAxVAxxLExLExΦ, and TVhynPxxLxxWxxxM, albeit with sequence variations in specific residues (Supplementary Figure S1B). The third Leu in the DeLLaΦLxYxV motif of VvDELLA3 is substituted by another aliphatic hydrophobic amino acid, Ala, while Val-40 (representing Φ) in VvDELLA1 and 2 is replaced by Gly-32 in VvDELLA3. Glu-36 (VvDELLA1) and Glu-47 (VvDELLA2) in this motif is also replaced by hydrophobic amino acid Gly-28 in VvDELLA3. The first Met in the MAxVAxxLExLExΦ motif of VvDELLA1 and VvDELLA2 is substituted by Leu in VvDELLA3, while Met-75 and Met-56 in VvDELLA2 and VvDELLA3, respectively, are replaced by Ile-64 in VvDELLA1. Thr-79 and Thr-90 in the TVhynPxxLxxWxxxM motif of VvDELLA1 and VvDELLA2, respectively, are substituted by non-polar Val-70 in VvDELLA3, whereas Val-80 and Val-91 in the former are replaced by Leu-71 in the latter. Tyr-82 (VvDELLA1) and Tyr-93 (VvDELLA2) are substituted by Cys-73 in VvDELLA3.

VvSLY1 genes and proteinsVvSLY1a and VvSLY1b are both 184 amino acid proteins, encoded by intron-less genes, located on chromosomes 7 and 18, respectively. VvSLY1a is a 20.2kDa protein, while VvSLY1b is 20.9kDa. Amino acid similarity between the VvSLY1 proteins is 60%, and identity to *Arabidopsis* and rice orthologues is 44 and 25% for VvSLY1a, and 51 and 28% for VvSLY1b, respectively (Fig. 1C). VvSLY1s alignment with AtSLY1 (McGinnis *et al.*, 2003) and OsGID2 (Sasaki *et al.*, 2003) shows two variable domains (one at the N-terminus and the other close to the C-terminus), conserved N-terminus F-box domains (52–80% sequence identity with AtSLY1 and OsGID2), and GGF (54–86% identity with AtSLY1 and OsGID2) and LSL (35–73% identity with AtSLY1 and OsGID2) domains located at the C-terminus (Supplementary Figure S1C).

**Fig. 2.**
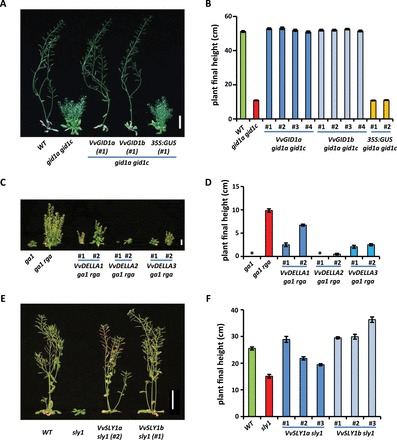
Grapevine GA signalling genes rescue the phenotype of corresponding *Arabidopsis* mutants. (A, C, E) Gross morphology of *gid1a-2 gid1c-2*, *ga1-3 rga-24*, and *sly1-10 Arabidopsis* mutants and representative transgenic plants transformed with VvGID1s, VvDELLLAs, and VvSLY1s, respectively. Bar: 5cm in (A), (E); 1cm in (C). Whole-plant pictures of VvGID1, VvDELLA, and VvSLY1 transformants were taken at 51, 70, and 60 days, respectively. (B, D, F) Average final plant heights of wild type (WT), mutant, and transgenic plants of (A), (C), and (E). The height of each individual transformant is significantly different from the corresponding mutant (*n* ≥ 8; *P* < 0.01). In contrast, the height of *35S:GUS gid1a gid1c* lines was not significantly different from *gid1a gid1c* (*n* ≥ 15). Parameters for VvGID1, VvDELLA, and VvSLY1 transformants were measured at 51, 81, and 87 days, respectively. Asterisk: zero plant height due to the lack of inflorescence stem elongation.

VvGID1s, VvDELLAs, and VvSLY1s are functional in transgenic *Arabidopsis* and in Y2H assays We tested the functions of these genes in *Arabidopsis* mutants. *35S:VvGID1a* and *35S:VvGID1b* were introduced separately into the *Arabidopsis gid1a gid1c* double mutant, which is a semi-dwarf with reduced fertility (decreased seed number per silique, and silique length) (Griffiths *et al.*, 2006). Expression of either VvGID1a or VvGID1b completely rescued all phenotypes of *gid1a gid1c* (Fig. 2A, B; Supplementary Figure S2A, B). *35S:VvDELLA1*, *35S:VvDELLA2*, and *35S:VvDELLA3* were transformed into the Arabidopsis *ga1-3 rga-24* mutant, which is a semi-dwarf (Dill and Sun, 2001). Expression of each VvDELLA reduced the plant height of *ga1-3 rga-24* (Fig. 2C, D), indicating that all three VvDELLAs were functional to rescue the *rga-24* defect. Similarly, *35S:VvSLY1a* and *35S:VvSLY1b* were introduced into the *Arabidopsis sly1-10* mutant, which is a semi-dwarf with reduced fertility (McGinnis *et al.*, 2003). Both VvSLY1a and VvSLY1b rescued the *sly1-10* mutant defects (Fig. 2E, F; Supplementary Figure S2C, D).

**Fig. 3.**
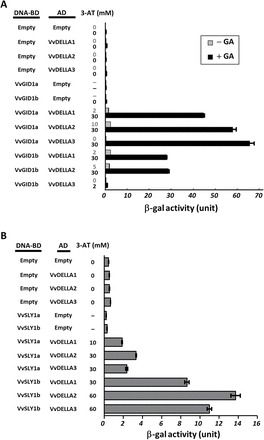
VvDELLAs interact with VvGID1s and VvSLY1s in Y2H assays. (A) Interaction between VvDELLAs and VvGID1s proceed in a GA-dependent manner. The addition of 100 µM GA_3_ to the medium enhanced GID1–DELLA interactions. (B) Interaction between VvDELLAs and VvSLY1s.

To further characterize the biochemical properties of VvGID1s, VvDELLAs, and VvSLY1s, we performed Y2H assays. Previous studies have shown that interactions between GID1 and DELLA from rice and *Arabidopsis* are enhanced in yeast cells in the presence of bioactive GAs (Ueguchi-Tanaka *et al.*, 2005; Griffiths *et al.*, 2006). We found that VvGID1a displayed GA-dependent binding to each of the three VvDELLAs (Fig. 3A). VvGID1b also interacted with VvDELLA1 and VvDELLA2 in a GA-dependent manner. Interestingly, VvDELLA–VvGID1a interactions were stronger, and VvGID1b did not interact with VvDELLA3, even in the presence of GA_3_. We also tested SLY1-DELLA interaction using Y2H assays. Similarly to *Arabidopsis* SLY1 (Dill *et al.*, 2004), both VvSLY1s interacted with all three VvDELLAs individually (Fig. 3B), although VvSLY1b showed stronger binding to VvDELLAs than VvSLY1a as indicated by the enhanced expression of the reporter genes (growth in higher 3-AT concentrations and higher β-gal activities). The results of the *in planta* functional assay and the Y2H assay indicate that these VvGID1s, VvDELLAs, and VvSLY1s function similarly to the *Arabidopsis* and rice orthologues in GA perception and signalling.

**Fig. 4.**
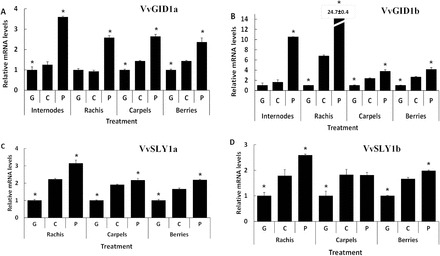
GA regulation of expression of *VvGID1a* (A), *VvGID1b* (B), *VvSLY1a* (C), and *VvSLY1b* (D) in selected tissues/organs of *V. vinifera* cv. Thompson Seedless. Tissues/organs were treated and sampled 6h after GA treatment and 102h after PAC treatment. Organs were dipped or sprayed until run-off with a single GA_3_ application (G), paclobutrazol (P), or Triton X-100 (C) treatment. The absolute mRNA levels of each gene were determined by qRT-PCR and normalized against *VvGAPDH*. Absolute gene expression in any organs/tissues are shown relative to the values for the GA treatment. The bars represent the mean ± SE of three biological repeats with two technical repeats each. Asterisks indicate values statistically different from their respective control (C) at *P* ≤ 0.05. Results were reproducible in successive growing seasons.

GA regulation of *VvGID1* and *VvSLY1* transcript levelsResults from several studies (Ueguchi-Tanaka *et al.*, 2005; Griffiths *et al.*, 2006; Voegele *et al.*, 2011) point to GA-induced feedback inhibition of *GID1* transcripts. To test whether similar regulation occurs in *Vitis*, we analysed the changes in expression of the *VvGID1* genes in different organs subjected to GA_3_ and PAC treatment regimes (Fig. 4A, B). Based on our results, it appears that: (i) with the exception of *VvGID1a* in rachis (Fig. 4A), GA_3_ treatment downregulated both *VvGID1* transcript levels, while PAC treatment enhanced their levels; (ii) the degree of change was lower in response to GA_3_ than in response to PAC, with the exception of *VvGID1b* levels in carpels and berries; (iii) generally, GA_3_ treatment resulted in greater downregulation in transcript levels of *VvGID1b* than *VvGID1a*. The most dramatic difference was in the rachis where GA_3_ treatment reduced *VvGID1b* mRNA levels by ~7-fold, but did not alter *VvGID1a* expression. (iv) Similarly, PAC treatment produced a greater increase of transcripts of *VvGID1b* than *VvGID1a*, resulting in a 3-, 10-, 1.5-, and 2-fold difference between both paralogues in internodes, rachis, carpels, and berries, respectively. Upregulation of *VvGID1a* and *VvGID1b* expression was maximal in the PAC-treated internodes (~3- and 6.5-fold, respectively).

*VvSLY1* expression displayed a similar pattern to that of *VvGID1*s (Fig. 4C, D). GA_3_ treatment downregulated transcript levels of both *VvSLY1* homologues, while PAC treatment increased their levels, compared to the respective control samples. Unlike *VvGID1*, the degree of regulation of expression of *VvSLY1a* and *VvSLY1b* was similar, and the effect of GA was more pronounced than that of PAC. There were only marginal differences when different organs were compared, and *VvSLY1b* seems to be unaffected by PAC treatment in carpels.

**Fig. 5.**
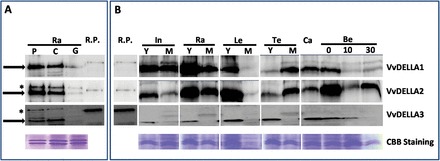
GA-induced degradation, and temporal and spatial profiles of VvDELLA proteins in *V. vinifera* cv. Thompson Seedless. Western blot analyses are shown of VvDELLA proteins in organs using affinity-purified, anti-VvDELLA polyclonal antibodies. Total protein extracted from organs across different developmental stages (full description given in Materials and Methods) was incubated with anti-VvDELLA polyclonal antibodies from rabbit. Recombinant full-length proteins (R.P.) (3.75ng each of VvDELLA1 and VvDELLA2, and 37.5ng of VvDELLA3) were used as controls. Coomassie Brilliant Blue (CBB)-stained proteins were used as loading controls. In all lanes except R.P., solid black arrows show the band of interest, and asterisked-bands indicate non-specific proteins detected by the anti-VvDELLA antibodies. Differences in sizes of R.P. and endogenous VvDELLA proteins result from V5 and 6xHis tags on the R.P. (A) The blot for GA-induced degradation of VvDELLA proteins contained total proteins extracted from young rachis (E-L 15) treated with 121 µM GA_3_ (G) for 6h, or 0.025% Triton X-100 (C) for 6h, or 0.8mM paclobutrazol (P) for 102h. (B) Temporal and spatial profiles of VvDELLA1, VvDELLA2, and VvDELLA3 in organs of cv. Thompson seedless. In, internodes; Ra, rachis; Le, leaves; Te, tendrils; Ca, carpels; Be, berries; 0, berries sampled at 2–3mm diameter (E-L 27); 10, berries sampled 10 day after E-L 27; 30, berries sampled 30 days after E-L 27; Y, young; M, mature. This figure is available in colour at *JXB* online.

GA regulation of VvDELLA proteins We did not find any distinct expression pattern of transcripts of *VvDELLA*s in response to GA_3_ or PAC treatments (Supplementary Figure S3). To test the effect of GA on the stability of VvDELLA proteins, we analysed levels of VvDELLA1, VvDELLA2, and VvDELLA3 in GA_3_- and PAC-treated rachises, internodes, and berries using antibodies specific for VvDELLA1, VvDELLA2, and VvDELLA3, separately. The immunoblot analyses showed that the level of all three VvDELLA proteins decreased with GA_3_ application, while PAC treatment appeared to only slightly increase the VvDELLA protein levels in the rachis (Fig. 5A; Supplementary Figure 4S).

**Fig. 6.**
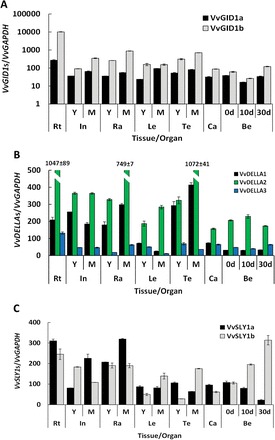
Spatial and temporal expression profiles in *V. vinifera* cv. Thompson seedless of *VvGID1a* and *VvGID1b* (A); *VvDELLA1*, *VvDELLA2*, and *VvDELLA3* (B); and *VvSLY1a* and *VvSLY1b* (C). Total RNA was extracted from pooled samples, and the absolute mRNA levels of each gene were determined by qRT-PCR and normalized against *VvGAPDH*. To ensure accurate quantitation of transcript levels, primers of similar efficiencies were used, and calibration curves determined from known copy numbers of single plasmids containing all qRT-PCR amplicons. The bars represent the mean ± SE of three biological repeats with two technical repeats each. In, internodes; Ra, rachis; Le, leaves; Te, tendrils; Ca, carpels; Be, berries; 0 d, berries sampled at 2–3mm diameter (E-L 27); 10 d, berries sampled 10 day after E-L 27; 30 d, berries sampled 30 days after E-L 27; Y, young; M, mature. The y-axis (expression) of panel (A) is presented with log values. This figure is available in colour at *JXB* online.

Spatial and temporal expression of GA signalling componentsHaving identified and functionally analysed the different paralogues of putative *VvDELLA*, *VvGID1*, and *VvSLY1* genes, we reasoned that they may exhibit different spatial and/or temporal expression profiles, which may be related to their function and/or to the quantities of endogenous bioactive GA species in these organs. Indeed, qRT-PCR analyses of the GA signalling components in selected organs revealed differential expression patterns (Fig. 6). *VvGID1a* and *VvGID1b* were redundantly expressed in all organs analysed, and their transcript levels were highest in the roots (Fig. 6A). However, *VvGID1a* expression was in general lower than *VvGID1b*, ranging from 1.5-fold lower in young berries (0 d) to 37-fold lower in roots. Interestingly, mRNA levels of *VvGID1s* increased in mature tissues compared to young tissues, except in berries where both *VvGID1* transcripts levels decreased 2-fold from fruit set to 10 DAF, followed by a 2- and 5-fold increase for *VvGID1a* and *VvGID1b*, respectively, from 10 to 30 DAF.

As shown in Fig. 6B, all three *VvDELLA*s were expressed in all tissues analysed, albeit at different quantities. Generally, *VvDELLA2* had the highest expression while *VvDELLA3* had the lowest expression in all organs, except berries in which *VvDELLA1* was lowest. Similarly to *VvGID1*s, roots contained relatively high levels of all three *VvDELLA*s, with *VvDELLA3* having the highest levels compared to all other organs. In rachis and tendrils, *VvDELLA1* and *VvDELLA2* mRNA levels increased in mature tissue. *VvDELLA3* expression also increased in mature rachis but decreased in mature tendrils. Intermediate levels of all *VvDELLA*s were detected in internodes, without a clear age-dependent pattern. Interestingly, similar to both *VvGID1*s, *VvDELLA3* expression decreased slightly from fruit set to 10 DAF followed by a 2-fold increase at 30 DAF. An inverse expression profile was observed for *VvDELLA2* over the same period of berry development.

As observed for *VvGID1*s and *VvDELLA*s, the expression of *VvSLY1a* and *VvSLY1b* (Fig. 6C) was high in the roots. Apart from roots, *VvSLY1a* had the highest expression in mature rachis, and *VvSLY1b* in berries at 30 DAF. Both paralogues showed distinctly opposing expression patterns during tissue development: whereas one paralogue was upregulated during tissue development, the other was either downregulated or unchanged. *VvSLY1a* levels increased with development of internodes and rachis (3- and 1.5-fold, respectively, compared to young tissues), remained unchanged during leaf development, and decreased as tendrils and berries developed. *VvSLY1b,* on the other hand, was upregulated during development of leaves, tendrils, and berries, representing 3- and 6-fold increases in the first two tissues when they matured, and a 3-fold increase in 30-d-old berries, compared to 0 d. *VvSLY1b* expression remained unchanged during rachis aging, and was downregulated as internodes matured.

Temporal and spatial profiles of VvDELLA proteins We carried out immunoblot analyses to examine whether different VvDELLA proteins show unique temporal or spatial expression patterns. In agreement with their mRNA expression profiles, all three VvDELLA proteins were present in the tissues analysed (Fig. 5B). Correlation between temporal transcript and protein profiles was evident for all VvDELLAs in tendrils and at the transition from carpels to berries, and also for VvDELLA1 and VvDELLA3 in leaves (Figs 5B and 6B). The protein profiles in all organs except berries also indicate that: (i) levels of VvDELLA1 and VvDELLA2 were lowest in mature leaves, and highest in young rachis; (ii) levels of VvDELLA1 and VvDELLA2 increased with tendril development, and decreased with leaf and rachis age; (iii) VvDELLA2 levels decreased whereas VvDELLA1 was unaffected by internode age; (iv) levels of VvDELLA3 considerably decreased as all organs aged.

Significant observations in VvDELLA protein profiles during berry development are: (i) levels of VvDELLA3 decreased steadily; (ii) compared to other VvDELLAs, levels of VvDELLA2 were induced dramatically at the transition from carpels to berries; (iii) VvDELLA1 and VvDELLA2 were lowest at 10 days of fruitlet development.

Endogenous GA quantities in developing grapevine organsThe fact that GA application reduced the expression of *VvGID1s* (Figs 4A and B), and caused degradation of VvDELLA proteins (Fig. 5A) prompted us to explore the hypothesis that the developmental expression and protein profiles of these genes may be determined, at least in part, by the endogenous GA quantities in the organs. To this end, the content of the bioactive GAs, GA_1_ and GA_4_, and their precursors and deactivation products were quantified (Table 1).

**Table 1.** Quantities of selected GA species in 13-hydroxylated and non-13-hydroxylated pathways in *V. vinifera* cv. Thomson seedless13-hydroxylated pathways are shown in the top half of the table and non-13-hydroxylated pathways in the bottom half. Values represent mean amounts of GA species (ng g^–1^ FW) determined in three biological replicates ± SE; n.d., undetected or could not be reliably quantified due to low abundance; n.q., detected, but could not be quantified due to co-migration of impurities or undetected internal standard (IS).

	Internode		Rachis		Leaves		Tendrils		Carpels	Berries		
GA species	Young	Mature	Young	Mature	Young	Mature	Young	Mature		0 d	10 d	30 d
GA_53_	2.6±0.3	n.d.	n.d.	n.d.	n.d.	n.d.	n.d.	n.d.	1.7±0.1	0.8±0.2	n.d.	n.d.
GA_44_	n.d.	n.d.	0.4±0.1	n.d.	n.d.	n.d.	n.d.	n.d.	1.1±0.0	0.8±0.1	0.1±0.0	n.d.
GA_19_	26.5±3.2	0.7±0.1	5.0±1.2	n.d.	24.7±0.5	n.d.	15.2±5.5	23.9±5.0	28.8±1.7	11.4±0.1	1.3±0.3	0.1±0.0
GA_20_	n.d.	n.d.	n.d.	n.d.	4.6±0.2	n.d.	0.7±0.1	n.d.	3.0±0.1	0.8±0.1	n.d.	n.d.
GA_29_	n.d.	n.d.	1.0±0.3	n.d.	4.3±0.4	n.d.	n.q.	n.d.	2.8±0.2	1.6±0.0	0.2±0.0	0.1±0.0
**GA**_**1**_	**n.d.**	**n.d.**	**n.d.**	**n.d.**	**n.d.**	**n.d.**	**2.9±1.2**	**0.9±0.2**	**2.8±0.2**	**0.7±0.2**	**0.4±0.1**	**n.d.**
GA_8_	2.2±0.1	n.d.	1.4±0.5	n.d.	5.6±0.8	1.1±0.5	n.q.	n.q.	9.8±1.0	14.8±0.8	1.0±0.2	0.2±0.0
GA_12_	1.8±0.1	n.d.	n.d.	n.d.	n.d.	n.d.	n.d.	n.d.	n.d.	n.d.	n.d.	n.d.
GA_24_	5.4±0.3	0.4±0.1	0.1±0.0	n.d.	0.9±0.1	n.q.	0.1±0.0	n.d.	n.q.	2.0±0.3	n.q.	n.d.
**GA**_**4**_	**0.6±0.1**	**0.2±0.0**	**0.1±0.0**	**n.d.**	**2.0±0.5**	**n.d.**	**2.1±0.5**	**n.d.**	**1.0±0.2**	**0.4±0.1**	**0.8±0.1**	**0.2±0.1**
GA_34_	0.8±0.1	n.d.	n.q.	n.q.	1.6±0.1	0.5±0.0	1.0±0.3	0.4±0.1	0.2±0.0	0.4±0.0	3.0±0.5	2.7±0.2

The 13-hydroxylation pathway, which leads to the biosynthesis of GA_1_ (Supplementary Figure S5), was characterized by the high levels of GA_19_ in all organs (Table 1). GA_1_ was present only in tendrils and carpels. Interestingly, GA_1_ levels reduced by 4-fold at fruit set, and further decreased as berries developed. GA_8_, the deactivation product of GA_1_, was detected in most organs, and showed a similar temporal profile as GA_1_. Notably, GA_8_ levels were elevated during carpel–berry transition, but markedly declined during berry development. The non-13-hydroxylation pathway that produces GA_4_ was characterized by relatively lower quantities of GA intermediates. GA_12_ was undetectable in all samples except young internodes. GA_4_ quantities in carpels decreased towards fruit set. It is important to note that from fruit set to 10 DAF, quantities of both GA_4_ and GA_34_ (the deactivation product of GA_4_) in berries increased. As berries developed, GA_34_ levels remained constant while GA_4_ levels decreased. In all but tendrils, carpels and 0 d berries, GA_4_ quantities were higher than GA_1_, whereas the opposite was true (except in tendrils and older berries) of GA_34_ and GA_8_. Generally, the levels of the different GA species either decreased or remained constant as tissues/organs developed.

### Effect of GA_3_ and PAC application on organ development

**Fig. 7.**
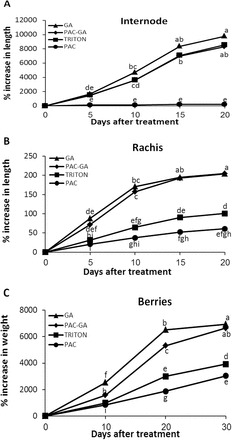
Altered response of organs of *V. vinifera* cv. Thompson seedless to GA3 and GA biosynthesis inhibitor (PAC) treatments. GA_3_ and PAC (0.8mM) were formulated in Triton X-100 (0.025%). Internodes and rachises were treated with 121 µM GA_3_, while berries were treated with 90 µM GA_3_. Tissues/organs were dipped or sprayed until run-off. Increase in size was monitored at specific time intervals. Young shoots and inflorescences with tightly packed flowers (stage 15, E-L 15, on the Modified Eichhorn and Lorenz system) were selected for internode and rachis experiments, respectively. Clusters with berries of 2–3mm diameter (E-L 27) were selected for berry experiments. (A) Lengths of new internodes arising after treating shoots. An increase in length of internode is expressed as per cent increase of initial length, which was assumed to be 0.5mm. (B) Changes in length of treated rachises, expressed as per cent increase of initial length. (C) Per cent increase in berry weight relative to mean weight at time of treatment (0 d). Data points with different letters indicate significantly different values according to the Tukey HSD LSMean test at α = 0.05 and 25 measurements, except for berries with 150 measurements.

Different grapevine organs exhibit markedly different responses to GA application (Weaver, 1958; Mullins *et al.*, 1992; Agüero *et al.*, 2000). To investigate whether such differential responses to GA_3_ application correlate with quantities of signalling components and/or endogenous GAs, we evaluated growth rates of internodes, rachis, and berries after GA_3_ and PAC treatments (Fig. 7). Our results show that all organs analysed displayed a GA-related growth response. However, different organs showed varying degrees of GA responses: rachis and berries responded more dramatically to GA_3_, while internodes had the greater response to PAC treatment. Compared to the control, PAC treatment resulted in marked (>40-fold) attenuation of internode elongation (Fig. 7A). About 40% of the PAC-treated shoot tips dropped, making it impossible to measure internode elongation on those shoots. GA treatment, on the other hand, resulted in an insignificant increase in internode growth (Fig. 7A). Both GA3 and PAC treatments resulted in ~2-fold change in rachis length (Fig. 7B), and in similar changes in berry weight (Fig. 7C). The GA_3_-treated berries obtained an elongated shape while PAC-treated and control berries presented a rounded shape. Furthermore, GA_3_ treatments partially or completely rescued the suppressive effect of PAC in all organs (PAC-GA treatments), confirming that the effect of PAC was GA associated.

## Discussion

The GA signalling cascade has been extensively described in a number of plant species. However, apart from VvDELLA1 (VvGAI1), characterized by Boss and Thomas (2002), little was known about the GA signalling components in grapevine, even though worldwide commercial cultivation of seedless table grapes involves intensive GA treatment for horticulture practices. This study is the first in-depth functional characterization of the major GA signalling components of grapevines.

### Structural and functional analyses of GA signalling genes

Functional studies in the *Arabidopsis gid1a gid1c* mutant (Fig. 2A, B; Supplementary Figure S2A, B) and Y2H analyses (Fig. 3A) indicate that both VvGID1s are active GA receptors. Because Thr-128 in GID1a is essential for the direct interaction with Φ of the DeLLaΦLxYxV motif (Murase *et al.*, 2008), it is likely that substituting this residue with the aromatic residue Tyr-128 in VvGID1b could account for the lower binding affinity of VvGID1b compared to VvGID1a with VvDELLA1 and VvDELLA2, as well as the lack of interaction between VvGID1b and VvDELLA3 in our Y2H tests. Expression of *AtGID1*s is downregulated by GA (Griffiths *et al.*, 2006). We observed a similar regulatory mechanism for both *VvGID1*s (Fig. 4A, B) in grapevine.

Since VvDELLA1 does not regulate berry enlargement (Boss and Thomas, 2002), we speculated that other DELLA family member(s) may modulate GA-related berry growth and responses. The two additional grapevine DELLA genes identified in this study, *VvDELLA2* and *VvDELLA3*, were biologically functional *in planta* (Fig. 2C, D), and in Y2H analyses (Fig. 3). Nonetheless, sequence variations in functionally important residues such as Ala-33, Gly-32, Val-70, Leu-71, and Cys-83 of VvDELLA3, predicted to be required for the direct interactions of VvDELLA with VvGID1 and/or stabilization of the VvGID1-VvDELLA complex, may account for the absence of interaction between VvDELLA3 and VvGID1b. These residues are critical in the GA-dependent interactions between *Arabidopsis* DELLA proteins and GID1s (Murase *et al.*, 2008). Similar to other model plants (Fu *et al.*, 2002; Itoh *et al.*, 2002; Griffiths *et al.*, 2006), degradation of all three VvDELLA proteins in response to GA treatment (Fig. 5A) further confirms their function as GA signalling repressors.

*VvDELLA2* mRNA and protein levels are highest in most tissues examined, suggesting that VvDELLA2 plays a major role in regulating GA responses at most developmental stages. *VvDELLA1* transcript and protein levels were relatively abundant in most tissues (e.g. roots, tendrils, and internodes), except in berries, consistent with a previous study (Boss and Thomas, 2002). However, contrary to the above-mentioned report, which did not identify *VvDELLA1* transcripts in berries, we detected low levels of *VvDELLA1* transcripts in berries of Thompson seedless (Fig. 6B). These differences could be due to difference in sensitivity between the detection techniques (qRT-PCR in the current study compared to RNA blotting in the former), or the varietal differences (sternospermocarpic cultivars in the present study compared to seeded cultivars in the former). The expression profile of VvDELLA1 suggests that VvDELLA1 plays a major role in most tissues, but only has a minor role during berry development. VvDELLA3 is expressed at much lower levels than the other two DELLAs, suggesting that DELLA3 plays a minor role in most tissues. This idea should be further tested by functional analysis *in planta* using a transgenic approach.

To the best of our knowledge, all analysed plants possess only one GA-specific F-box protein in the SLY1/GID2 family (McGinnis *et al.*, 2003; Sasaki *et al.*, 2003). Thus, the presence of two biologically functional paralogues of GA-specific F-box proteins (Fig. 2E, F; and Fig. 3B) in grape vine is unique. These paralogues may have arisen from a duplication event, and their maintenance in the genome may be due to differential specificity of interaction with the various VvDELLA-VvGID1 complexes. Such a possibility is supported by the differences in DELLA–SLY interactions demonstrated in our Y2H analyses. Downregulation of *VvSLY1* expression by GA application (Fig. 4C, D) is supported by the negative feedback regulation of *Arabidopsis SLY1* by GA_3_ (Dill *et al.*, 2004). Our results suggest that temporal expression profiles of the *VvSLY1* genes *in planta* may be regulated by the endogenous bioactive GA quantities as well. Moreover, a correlation between the temporal expression pattern of each *VvSLY1* homologue and endogenous GA levels may be indicative of the role each paralogue plays in GA-related organ development. We therefore suggest that *VvSLY1a* may be playing a central role in GA-regulated growth of internodes and rachis, while *VvSLY1b* may be important for leaf and tendril development, as well as carpel development, because its expression increased after fruit set as the bioactive GA_1_ levels in the organ decreased.

Correlation between levels of endogenous GAs and GA signalling componentsSimilar to *Arabidopsis* and other species (Dill *et al.*, 2001; Itoh *et al.*, 2002; Griffiths *et al.*, 2006), we have shown that in grapevine application of GA_3_ downregulates *VvGID1* transcripts and results in degradation of VvDELLA proteins (Fig. 4A, B; Fig. 5A). In light of the above, it was important to quantify endogenous GAs in grapevine organs, and determine how they correlate with levels of the signalling components in these organs. The results of our GA quantitative analysis, which show a general decrease in GA species during organ development (Table 1), is consistent with that of wine grapes (Boll *et al.*, 2009), avocado (Raviv *et al.*, 1987), and other species (Itoh *et al.*, 1999; Kaneko *et al.*, 2003). Interestingly, although GA_19_ levels are higher than its corresponding GA_24_ counterpart in the non-13-hydroxylated pathway, GA_4_ was higher than GA_1_ in most organs. This could be due to higher turnover of GA_1_, supported also by higher levels of GA_8_ than GA_34_ in these organs.

Higher quantities of GA_4_ in internode, rachis, leaves, and berries suggest that this molecule is the major bioactive GA promoting growth of these organs. In contrast and consistent with seeded wine grapes (Perez *et al.*, 2000; Boll *et al.*, 2009; Giacomelli *et al.*, 2013), carpels contain relatively high levels of GA_1_ and most of its precursors, suggesting a unique role of GA_1_ in regulating flower development and fruit set. A unique ‘convex’ profile of GA_4_ in berries should be highlighted, where levels of GA_4_ are increasing in a certain period of berry development (0–10 DAF) rather than decreasing as is the case for other ageing tissues. This profile may reflect the transition between sources of production of GA_4_ at fruit set, and the unique development of the stenospermocarpic berry. We speculate that: (i) prior to anthesis, anthers probably serve as the main source of bioactive GAs (GA_1_ and low levels of GA_4_), and supply from this source decreases at bloom as stamens fall and fruit set commences; (ii) after fruit set, seeds serve as the main source of GA_4_ accumulation, as shown in other plants (MacMillan, 2001). GA_4_ increases as seeds develop, and in stenospermocarpic varieties, peaks just before abortion of endosperm (which occurs at about 14 DAF); (iii) After abortion, GA_4_ supply from the seed rudiments is decreased.

Endogenous bioactive GAs regulate temporal profiles of *VvGID1* transcripts in a negative feedback fashion, while the GA regulation of temporal profiles of VvDELLA proteins appears to be organ-specific. The expected inverse correlation was observed in tendrils and berries for both VvDELLA1 and VvDELLA2. As bioactive GA quantities decrease during transition from carpel to fruitlet (fruit set), there is a parallel increase in VvDELLA2 accumulation. The subsequent increase in GA_4_ levels as fruitlets develop, prior to endosperm abortion, correlates with the decrease in VvDELLA1 and VvDELLA2 quantities during the same period. Reduction in GA_4_ quantities after abortion is mirrored by the corresponding significant accumulation of VvDELLA2, and a slight increase of VvDELLA1. This connection between developmental transitions throughout berry development, GA levels, and the VvDELLA protein quantities, may indicate the functional importance of these VvDELLA paralogues in the regulation of GA-mediated berry development.

In all other organs, however, the decrease in endogenous bioactive GA detected in older tissue, compared with a young tissue, was accompanied by decreased levels of VvDELLAs. These results are consistent with a previous study (Chandler *et al.*, 2002) showing that bioactive GA levels in developing barley leaves correlate with DELLA protein levels. In these organs, it is possible that DELLA proteins may play a more important role in the rapidly growing tissues to regulate growth dynamically. As these organs mature and growth rate declines, DELLAs may not be required to regulate growth.

### Organ responses to GA_3_ and PAC depend on both GA signalling components and endogenous GA levels

It can be assumed that a young developing organ with relatively high VvDELLA protein quantities will have a greater response to GA treatment. To test this idea, we compared VvDELLA levels to the response of growing organs to GA_3_ application. The sum of all three VvDELLA proteins was highest in young rachis, intermediate in young berries, and lowest in young internodes (Fig. 5B). Consistent with our hypothesis, and compared to their respective controls, GA_3_ application resulted in a 2-fold increase in rachis and berry size, while a similar treatment did not affect internode length (Fig. 7A). PAC treatments, on the other hand, resulted in a 40-fold reduction in internode length, while producing ~1.5-fold reduction in rachis length and berry weight.

High vegetative vigour in grapevines was associated with high endogenous GA levels (Lavee, 1986). Accordingly, it is expected that a minimal amount of GA is required to fulfil the growth potential of an organ under given conditions and allow maximal size enhancement. At such optimal levels of endogenous GA, high response to PAC treatment is expected, and a much subdued or zero response to GA application. Conversely, organs with levels of endogenous bioactive GA that are below this optimum will exhibit a high response to GA treatment, and show little or no response to PAC treatment, depending on the difference between the level of endogenous GA and that required for maximal growth. The data reported in the current study (Table 1; Fig. 7) supports the above scenario and suggest that different organs require different quantities of bioactive GA for maximal size enhancement. The 0.6ng g^–1^ FW of GA_4_ in young internodes was enough to elicit maximal growth in the internodes, as evidenced by the non-responsiveness of the internodes to GA_3_ application, and the huge response to PAC treatment. In contrast, in young berries, 0.4ng g^–1^ FW GA_4_ (berries at 0 d), and even 0.8ng g^–1^ FW (berries at 10 d), were insufficient to ensure development to maximal berry size. Hence, both GA_3_ and PAC treatments resulted in significant changes in berry size. Similar to our results, GA_3_-related berry enlargement of Thompson seedless has been widely reported (Harrell and Williams, 1987; Ben-Tal, 1990). In the case of young rachis, 0.1ng g^–1^ FW GA_4_ was only sufficient to produce limited growth, and GA_3_ application had a significant contribution. Effective GA-related rachis elongation is widely employed by viticulturists to reduce compactness within the cluster and prevent berry rot (Weaver and Pool, 1965; Dokoozlian and Peacock, 2001).

## Supplementary material

Supplementary data can be found at *JXB* online.

Supplementary Table S1. Primers used for gene isolation and gene expression analyses by qRT-PCR.

Supplementary Table S2. Primers used for cloning genes to Y2H vectors.

Supplementary Table S3. Amount of deuterated GA species used as Internal Standard (IS) mix during extraction of endogenous GAs in different organs of grapes.

Supplementary Table S4. GA signalling genes of *V. vinifera* cv. Thompson seedless.

Supplementary Figure S1. Amino acid sequence alignment of major GA signalling genes.

Supplementary Figure S2. Grapevine GA signalling genes rescue silique length and fertility defects of corresponding *Arabidopsis* mutants.

Supplementary Figure S3. GA regulation of expression of *VvDELLA1*, *VvDELLA2*, and *VvDELLA3*.

Supplementary Figure S4. GA_3_-induced degradation of VvDELLA proteins in cv. Thompson seedless.

Supplementary Figure S5. Schematic representation of major GA metabolism pathways in plants.

## Funding

This research was supported by the United States-Israel Binational Agricultural Research and Development Fund (BARD grant no. IS-4018-07 to EO, AL, and T-PS), and the USDA National Institute of Food and Agriculture (Research Initiative Competitive Grant no. 2010-65116-2046 and 2014-67013-21548 to T-PS).

## Supplementary Material

Supplementary Data

## References

[CIT0001] Acheampong AK Rotman A Zaheng C Keren A Halaly T Crane O Ogrodovitch A Or E 2010 A method for isolating total RNA from mature buds and other woody tissues of Vitis Vinifera. In: Delrot S Or E Babaresco L Grando S, eds. Methodologies and results in grapevine research. The Netherlands: Springer, 301–307.

[CIT0002] Agüero C Vigliocco A Abdala G Tizio R 2000 Effect of gibberellic acid and uniconazol on embryo abortion in the stenospermocarpic grape cultivars Emperatriz and Perlon. Plant Growth Regulation 30, 9–16.

[CIT0003] Ben-Tal Y 1990 Effects of gibberellin treatments on ripening and berry drop from Thompson Seedless grapes. American Journal of Enology and Viticulture 41, 142–146.

[CIT0004] Boll S Lange T Hofmann H Schwappach P 2009 Correspondence between gibberellin-sensitivity and pollen tube abundance in different seeded vine varieties. Mitteilungen Klosterneuburg 59, 129–133.

[CIT0005] Boss PK Thomas MR 2002 Association of dwarfism and floral induction with a grape ‘green revolution’ mutation. Nature 416, 847–850. 11976683 10.1038/416847a

[CIT0006] Chandler PM Marion-Poll A Ellis M Gubler F 2002 Mutants at the *Slender1* Locus of Barley cv Himalaya. Molecular and physiological characterization. Plant Physiology 129, 181–190. 12011349 10.1104/pp.010917 PMC155882

[CIT0007] Chundawat BS Takahashi E Nagasawa K 1971 Effect of gibberellic acid, B-nine and keratin on fruit set, parthenocarpy and quality of kyoho grapes Journal of Japanese Society of Horticultural Sciences 40, 5–9.

[CIT0008] Conde C Silva P Fontes N Dias ACP Tavares RM Sousa MJ Agasse A Delrot S Geros H 2007 Biochemical changes throughout grape berry development and fruit and wine quality. Food 1, 1–22.

[CIT0009] Coombe B 1995 Adoption of a system for identifying grapevine growth stages. Australian Journal of Grape and Wine Research 1, 100–110.

[CIT0010] Coombe BG 1960 Relationship of growth and development to changes in sugars, auxins, and gibberellins in fruit of seeded and seedless varieties of Vitis vinifera. Plant Physiology 35, 241–250. 16655336 10.1104/pp.35.2.241 PMC405950

[CIT0011] Dennis Jr FG Nitsch JP 1966 Identification of gibberellins A4 and A7 in ammature apple seeds. Nature 211, 781–782.

[CIT0012] Dereeper A Guignon V Blanc G 2008 Phylogeny.fr: robust phylogenetic analysis for the non-specialist. Nucleic Acids Research 36, 465–469. 10.1093/nar/gkn180 PMC2447785 18424797

[CIT0013] Dill A Jung HS Sun T 2001 The DELLA motif is essential for gibberellin-induced degradation of RGA. Proceedings of the National Academy of Sciences, USA 98, 14162–14167. 10.1073/pnas.251534098 PMC61185 11717468

[CIT0014] Dill A Sun T-P 2001 Synergistic derepression of gibberellin signaling by removing RGA and GAI function in Arabidopsis thaliana. Genetics 159, 777–785. 11606552 10.1093/genetics/159.2.777 PMC1461816

[CIT0015] Dill A Thomas SG Hu J Steber CM Sun T 2004 The Arabidopsis F-box protein SLEEPY1 targets gibberellin signaling repressors for gibberellin-induced degradation. The Plant Cell 16, 1392–1405. 15155881 10.1105/tpc.020958 PMC490034

[CIT0016] Dokoozlian NK Peacock WL 2001 Gibberellic acid applied at bloom reduces fruit set and improves size of ‘Crimson Seedless’ table grapes. HortScience 36, 706–709.

[CIT0017] Fleet CM Sun T 2005 A DELLAcate balance: the role of gibberellin in plant morphogenesis. Current Opinion in Plant Biology 8, 77–85. 15653404 10.1016/j.pbi.2004.11.015

[CIT0018] Fu X Richards DE Ait-ali T Hynes LW Ougham H Peng J Harberd NP 2002 Gibberellin-mediated proteasome-dependent degradation of the barley DELLA protein SLN1 repressor. The Plant Cell 14, 3191–3201. 12468736 10.1105/tpc.006197 PMC151211

[CIT0019] Giacomelli L Rota-Stabelli O Masuero D Acheampong AK Moretto M Caputi L Vrhovsek U Moser C 2013 Gibberellin metabolism in Vitis vinifera L. during bloom and fruit-set: functional characterization and evolution of grapevine gibberellin oxidases. Journal of Experimental Botany 64, 4403–4419. 24006417 10.1093/jxb/ert251 PMC3808322

[CIT0020] Griffiths J Murase K Rieu I 2006 Genetic characterization and functional analysis of the GID1 gibberellin receptors in Arabidopsis. The Plant Cell 18, 3399–3414. 17194763 10.1105/tpc.106.047415 PMC1785415

[CIT0021] Harrell DC Williams LE 1987 The influence of girdling and gibberellic acid application at fruitset on Ruby Seedless and Thompson Seedless grapes. American Journal of Enology and Viticulture 38, 83–88.

[CIT0022] Hirano K Ueguchi-Tanaka M Matsuoka M 2008 GID1-mediated gibberellin signaling in plants. Trends in Plant Science 13, 192–199. 18337155 10.1016/j.tplants.2008.02.005

[CIT0023] Hu J Mitchum MG Barnaby N 2008 Potential sites of bioactive gibberellin production during reproductive growth in Arabidopsis. The Plant Cell 20, 320–336. 18310462 10.1105/tpc.107.057752 PMC2276448

[CIT0024] Itoh H Tanaka-Ueguchi M Kawaide H Chen XB Kamiya Y Matsuoka M 1999 The gene encoding tobacco gibberellin 3 β-hydroxylase is expressed at the site of GA action during stem elongation and flower organ development. The Plant Journal 20, 15–24. 10571861 10.1046/j.1365-313x.1999.00568.x

[CIT0025] Itoh H Ueguchi-Tanaka M Sato Y Ashikari M Matsuoka M 2002 The gibberellin signaling pathway is regulated by the appearance and disappearance of SLENDER RICE1 in nuclei. The Plant Cell 14, 57–70. 11826299 10.1105/tpc.010319 PMC150551

[CIT0026] Iwahori S Weaver RJ Pool RM 1967 Gibberellin-like Activity in Berries of Seeded and Seedless Tokay Grapes. Plant Physiology 43, 333–337. 16656767 10.1104/pp.43.3.333 PMC1086842

[CIT0027] Jaillon O Aury JM Noel B 2007 The grapevine genome sequence suggests ancestral hexaploidization in major angiosperm phyla. Nature 449, 463–467. 17721507 10.1038/nature06148

[CIT0028] Kaneko M Itoh H Inukai Y Sakamoto T Ueguchi-Tanaka M Ashikari M Matsuoka M 2003 Where do gibberellin biosynthesis and gibberellin signaling occur in rice plants? The Plant Journal 35, 104–115. 12834406 10.1046/j.1365-313x.2003.01780.x

[CIT0029] Karimi M Inzé D Depicker A 2002 GATEWAY (TM) vectors for Agrobacterium-mediated plant transformation. Trends in Plant Science 7, 193–195. 11992820 10.1016/s1360-1385(02)02251-3

[CIT0030] Kaufman PB Ghosheh NS Nakosteen L Pharis RP Durley RC Morf W 1976 Analysis of native gibberellins in the internode, nodes, leaves, and inflorescence of developing Avena plants. Plant Physiology 58, 131–134. 16659632 10.1104/pp.58.2.131 PMC542197

[CIT0031] Kuderova A Nanak E Truksa M Brzobohaty B 1999 Use of rifampicin in T7 RNA polymerase-driven expression of a plant enzyme: rifampicin improves yield and assembly. Protein Expression and Purification 16, 405–409. 10425161 10.1006/prep.1999.1079

[CIT0032] Lavee S 1960 Effect of gibberellic acid on seeded grapes. Nature 185, 395.13852645

[CIT0033] Lavee S 1986 Usefullness of growth regulators for controlling vine growth and improving grape quality in intensive vineyards. Acta Horticulturae 206, 89–108.

[CIT0034] Lu G Moriyama EN 2004 Vector NTI, a balanced all-in-one sequence analyses suite. Briefings in Bioinformatics 5, 378–388. 15606974 10.1093/bib/5.4.378

[CIT0035] MacMillan J 2001 Occurrence of gibberellins in vascular plants, fungi, and bacteria. Journal of Plant Growth Regulation 20, 387–442. 11986764 10.1007/s003440010038

[CIT0036] McGinnis KM Thomas SG Soule JD Strader LC Zale JM Sun T Steber CM 2003 The Arabidopsis SLEEPY1 gene encodes a putative F-box subunit of an SCF E3 ubiquitin ligase. The Plant Cell 15, 1120–1130. 12724538 10.1105/tpc.010827 PMC153720

[CIT0037] Mosesian RM Nelson KE 1968 Effect on’Thompson Seedless’ fruit of gibberellic acid bloom sprays and double girdling. American Journal of Enology and Viticulture 19, 37–46.

[CIT0038] Mullins MG Bouquet A Williams LE 1992 Biology of the grapevine. Cambridge University Press.

[CIT0039] Murase K Hirano Y Sun T Hakoshima T 2008 Gibberellin-induced DELLA recognition by the gibberellin receptor GID1. Nature 456, 459–463. 19037309 10.1038/nature07519

[CIT0040] Nakajima M Shimada A Takashi Y 2006 Identification and characterization of Arabidopsis gibberellin receptors. The Plant Journal 46, 880–889. 16709201 10.1111/j.1365-313X.2006.02748.x

[CIT0041] Nijjar GS Bhatia GG 1969 Effect of gibberellic acid and para-chlorophenoxyacetic acid on cropping and quality of Anab-e-Shahi grapes. Journal of Horticultural Sciences and Biotechnology 44, 91–95.

[CIT0042] Perez FJ Viani C Retamales J 2000 Bioactive gibberellins in seeded and seedless grapes: identification and changes in content during berry development. American Journal of Enology and Viticulture 51, 315–318.

[CIT0043] Plackett AR Powers SJ Fernandez-Garcia N 2012 Analysis of the developmental roles of the Arabidopsis gibberellin 20-oxidases demonstrates that GA20ox1, -2, and -3 are the dominant paralogs. The Plant Cell 24 941–960. 22427334 10.1105/tpc.111.095109 PMC3336139

[CIT0044] Raviv M Reuveni O Goldschmidt EE 1987 The physiological basis for loss of rootability with age in avocado seedlings. Tree Physiology 3, 115–122. 14975824 10.1093/treephys/3.2.115

[CIT0045] Reid KE Olsson N Schlosser J Peng F Lund ST 2006 An optimized grapevine RNA isolation procedure and statistical determination of reference genes for real-time RT-PCR during berry development. BMC Plant Biology 6, 27–37. 17105665 10.1186/1471-2229-6-27 PMC1654153

[CIT0046] Sasaki A Itoh H Gomi K 2003 Accumulation of phosphorylated repressor for gibberellin signaling in an F-box mutant. Science 299, 1896–1898. 12649483 10.1126/science.1081077

[CIT0047] Silverstone AL Ciampaglio CN Sun T-P 1998 The Arabidopsis RGA gene encodes a transcriptional regulator repressing the gibberellin signal transduction pathway. The Plant Cell 10, 155–169. 9490740 10.1105/tpc.10.2.155 PMC143987

[CIT0048] Srinivasan C Mullins MG 1981 Physiology of flowering in the grapevine - a review. American Journal of Enology and Viticulture 32, 47–63.

[CIT0049] Steber CM Cooney SE McCourt P 1998 Isolation of the GA-response mutant sly1 as a suppressor of ABI1-1 in Arabidopsis thaliana. Genetics 149, 509–521. 9611170 10.1093/genetics/149.2.509 PMC1460187

[CIT0050] Sun T 2011 The Molecular mechanism and evolution of the GA-GID1-DELLA signaling module in plants. Current Biology 21, R338–R345. 21549956 10.1016/j.cub.2011.02.036

[CIT0051] Thomas SG Phillips AL Hedden P 1999 Molecular cloning and functional expression of gibberellin 2-oxidases, multifunctional enzymes involved in gibberellin deactivation. Proceedings of the National Academy of Sciences, USA 96, 4698–4703. 10.1073/pnas.96.8.4698 PMC16395 10200325

[CIT0052] Thompson JD Higgins DG Gibson TJ 1994 CLUSTAL W: improving the sensitivity of progressive multiple sequence alignment through sequence weighting, position-specific gap penalties and weight matrix choice. Nucleic Acids Research 22, 4673–4680. 7984417 10.1093/nar/22.22.4673 PMC308517

[CIT0053] Ueguchi-Tanaka M Ashikari M Nakajima M 2005 GIBBERELLIN INSENSITIVE DWARF1 encodes a soluble receptor for gibberellin. Nature 437, 693–698. 16193045 10.1038/nature04028

[CIT0054] Velasco R Zharkikh A Troggio M 2007 A high quality draft consensus sequence of the genome of a heterozygous grapevine variety. PLoS ONE 2, e1326. 18094749 10.1371/journal.pone.0001326 PMC2147077

[CIT0055] Voegele A Linkies A Müller K Leubner-Metzger G 2011 Members of the gibberellin receptor gene family GID1 (GIBBERELLIN INSENSITIVE DWARF1) play distinct roles during Lepidium sativum and Arabidopsis thaliana seed germination. Journal of Experimental Botany 62, 5131–5147. 21778177 10.1093/jxb/err214 PMC3193015

[CIT0056] Wang W Vignani R Scali M Cresti M 2006 A universal and rapid protocol for protein extraction from recalcitrant plant tissues for proteomic analysis. Electrophoresis 27, 2782–2786. 16732618 10.1002/elps.200500722

[CIT0057] Weaver RJ 1958 Effect of gibberellic acid on fruit set and berry enlargement in seedless grapes of *Vitis vinifera* . Nature 181, 851–852.13526698

[CIT0058] Weaver RJ Pool RM 1965 Bloom spraying with gibberellin loosens clusters of Thompson Seedless grapes. California Agriculture 19, 14–15.

[CIT0059] Wolf EEH Loubser JT 1992 Gibberellic acid levels and quality effects of gibberellic acid in treated table grapes. The South African Journal for Enology and Viticulture 13, 57–63.

[CIT0060] Yamaguchi S 2008 Gibberellin metabolism and its regulation. Annual Review of Plant Biology 59, 225–251. 10.1146/annurev.arplant.59.032607.092804 18173378

[CIT0061] Yamamoto Y Hirai T Yamamoto E Kawamura M Sato T Kitano H Matsuoka M Ueguchi-Tanaka M 2010 A rice *gid1* suppressor mutant reveals that Gibberellin is not always required for interaction between its receptor, GID1, and DELLA proteins. The Plant Cell 22, 3589–3602. 21098733 10.1105/tpc.110.074542 PMC3015124

