# Supplementary material for: Functional characterization and developmental expression profiling of gibberellin signalling components in Vitis vinifera
Source: J Exp Bot. 2015 Jan 14;66(5):1463–76. doi: 10.1093/jxb/eru504 (PMC4339604; doi:10.1093/jxb/eru504)
Supplement: Supplementary Data [file supp_66_5_1463__index.html]

Functional characterization and developmental expression profiling of gibberellin signalling components in Vitis vinifera — Supplementary Data 

# Functional characterization and developmental expression profiling of gibberellin signalling components in *Vitis vinifera*

## Supplementary Data

Data files

**Files in this Data Supplement:**

- Supplementary Data - Supplementary Data
