# Supplementary material for: Functional characterization and developmental expression profiling of gibberellin signalling components in Vitis vinifera
Source: J Exp Bot. 2015 Jan 14;66(5):1463–76. doi: 10.1093/jxb/eru504 (PMC4339604; doi:10.1093/jxb/eru504)
Supplement: Supplementary Data [file supp_eru504_jexbot137315_file001.pdf]

**Manuscript Title:** Functional Characterization and Developmental Expression Profiling of Gibberellin Signaling Components in *Vitis vinifera*

Atiako Kwame Acheampong, Jianhong Hu, Ariel Rotman, Chuanlin Zheng, Tamar Halaly, Yumiko Takebayashi, Jikumaru Yusuke, Yuji Kamiya, Amnon Lichter, Tai-Ping Sun, Etti Or

S1A

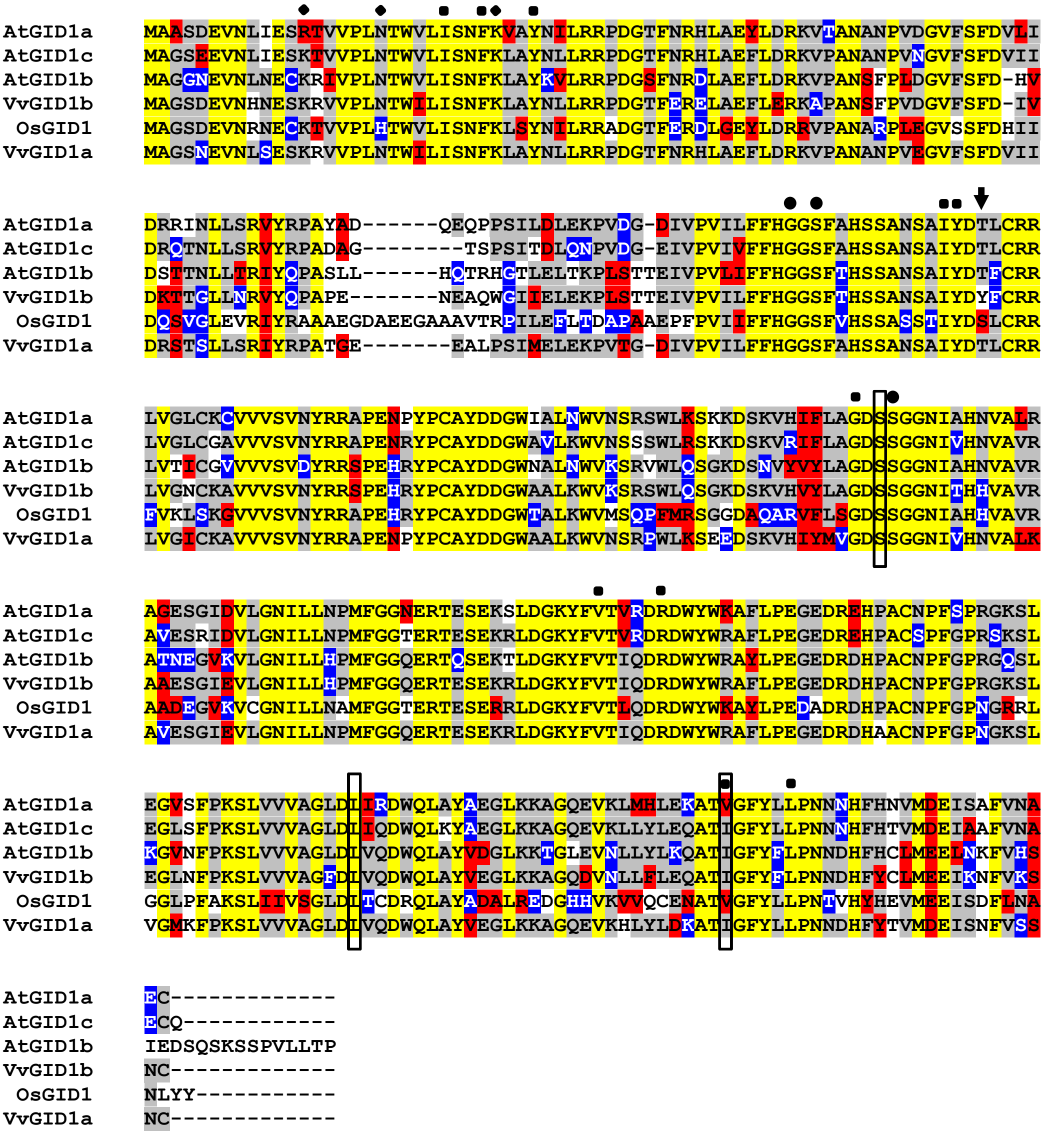
AtGID1a MAASDEVNLIESRTVVPLNTWVLISNFKVAYNILRRPDGTFNRHLAEYLDRKVTANANPVDGVFSFDVLI
AtGID1c MAGSEEVNLIESKTVVPLNTWVLISNFKLAYNLLRRPDGTFNRHLAEFLDRKVPANANPVNGVFSFDVII
AtGID1b MAGGNEVNLNECKRIVPLNTWVLISNFKLAYKVLRRPDGSFNRDLAEFLDRKVPANSFPLDGVFSFD-HV
VvGID1b MAGSDEVNHNESKRVVPLNTWILISNFKLAYNLLRRPDGTFERELAEFLERKAPANSFPVDGVFSFD-IV
OsGID1 MAGSDEVNRNECKTVVPLHTWVLISNFKLSYNILRRADGTFERDLGEYLDRRVPANARPLEGVSSFDHII
VvGID1a MAGSNEVNLSESKRVVPLNTWILISNFKLAYNLLRRPDGTFNRHLAEFLDRKVPANANPVEGVFSFDVII
AtGID1a DRRINLLSRVYRPAYAD------QEQPPSILDLEKPVDG-DIVPVILFFHGGSFAHSSANSAIYDTLCRR
AtGID1c DRQTNLLSRVYRPADAG--------TSPSITDLQNPVDG-EIVPVIVFFHGGSFAHSSANSAIYDTLCRR
AtGID1b DSTTNLLTRIYQPASLL------HQTRHGTLELTKPLSTTEIVPVLIFFHGGSFTHSSANSAIYDTFCRR
VvGID1b DKTTGLLNRVYQPAPE-------NEAQWGIIELEKPLSTTEIVPVILFFHGGSFTHSSANSAIYDYFCRR
OsGID1 DQSVGLEVRIYRAAAEGDAEEGAAAVTRPILEFLTDAPAAEPFPVIIFFHGGSFVHSSASSTIYDSLCRR
VvGID1a DRSTSLLSRIYRPATGE-------EALPSIMELEKPVTG-DIVPVILFFHGGSFAHSSANSAIYDTLCRR
AtGID1a LVGLCKCVVVSVNYRRAPENPYPCAYDDGWIALNWVNSRSWLKSKKDSKVHIFLAGDSSGGNIAHNVALR
AtGID1c LVGLCGAVVVSVNYRRAPENRYPCAYDDGWAVLKWVNSSSWLRSKKDSKVRIFLAGDSSGGNIVHNVAVR
AtGID1b LVTICGVVVVSVDYRRSPEHRYPCAYDDGWNALNWVKSRVWLQSGKDSNVYVYLAGDSSGGNIAHNVAVR
VvGID1b LVGNCKAVVVSVNYRRSPEHRYPCAYDDGWAALKWVKSRSWLQSGKDSKVHVYLAGDSSGGNITHHVAVR
OsGID1 FVKLSKGVVVSVNYRRAPEHRYPCAYDDGWTALKWVMSQPFMRSGGDAQARVFLSGDSSGGNIAHHVAVR
VvGID1a LVGICKAVVVSVNYRRAPENPYPCAYDDGWAALKWVNSRPWLKSEEDSKVHIYMVGDSSGGNIVHNVALK
AtGID1a AGESGIDVLGNILLNPMFGGNERTESEKSLDGKYFVTVRDRDWYWKAFLPEGEDREHPACNPFSPRGKSL
AtGID1c AVESRIDVLGNILLNPMFGGTERTESEKRLDGKYFVTVRDRDWYWRAFLPEGEDREHPACSPFGPRSKSL
AtGID1b ATNEGVKVLGNILLHPMFGGQERTQSEKTLDGKYFVTIQDRDWYWRAYLPEGEDRDHPACNPFGPRGQSL
VvGID1b AAESGIEVLGNILLHPMFGGQERTESEKRLDGKYFVTIQDRDWYWRAFLPEGEDRDHPACNPFGPRGKSL
OsGID1 AADEGVKVCGNILLNAMFGGTERTESERRLDGKYFVTLQDRDWYWKAYLPEDADRDHPACNPFGPNGRRL
VvGID1a AVESGIEVLGNILLNPMFGGQERTESEKRLDGKYFVTIQDRDWYWRAFLPEGEDRDHAACNPFGPNGKSL
AtGID1a EGVSFPKSLVVVAGLDLIRDWQLAYAEGLKKAGQEVKLMHLEKATVGFYLLPNNNHFHNVMDEISAFVNA
AtGID1c EGLSFPKSLVVVAGLDLIQDWQLKYAEGLKKAGQEVKLLYLEQATIGFYLLPNNNHFHTVMDEIAAFVNA
AtGID1b KGVNFPKSLVVVAGLDLVQDWQLAYVDGLKKTGLEVNLLYLKQATIGFYFLPNNDHFHCLMEELNKFVHS
VvGID1b EGLNFPKSLVVVAGFDLVQDWQLAYVEGLKKAGQDVNLLFLEQATIGFYFLPNNDHFYCLMEEIKNFVKS
OsGID1 GGLPFAKSLIIVSGLDLTCDRQLAYADALREDGHHVKVVQCENATVGFYLLPNTVHYHEVMEEISDFLNA
VvGID1a VGMKFPKSLVVVAGLDLVQDWQLAYVEGLKKAGQEVKHLYLDKATIGFYLLPNNDHFYTVMDEISNFVSS
AtGID1a EC-------------
AtGID1c ECQ------------
AtGID1b IEDSQSKSSPVLLTP
VvGID1b NC-------------
OsGID1 NLYY-----------
VvGID1a NC-------------

S1B

```
   AtRGA    MKRDHHQFQGRLSNHGTSSSSSS-----ISKDKMMMVKKEEDGGGNMDDELLAVLGYKVRSSEMAEVALK
VvDELLA1    MKREYHHPHHPTCSTSPTGKG-----------KMWDADPQQDAG---MDELLAVLGYNVKASDMAEVAQK
VvDELLA2    MKRDLLDGCGGGGGGGGGKGEEGSSMVSAKGKMWDDGQQQDAG---MDELLAVLGYNVRSSDMVDVAQK
VvDELLA3    --------MGPYDSAISTGSSTS-------------S-PSSAAKPSDIDGLLAGAGYKVRSTELHQVAQR

   AtRGA    LEQLETMMSNVQEDGLSHLATDTVHYNPSELYSWLDNMLSELNP---------------PPLPAS----
VvDELLA1    LEQLEEVIVNAQEDGLSHLASETVHYNPSDLSNWLGSMLSEFNP--------TPNCALDNPFLPP-----
VvDELLA2    LEQLEMVMGNAQEDGISHLSSGTVHYNPSDLSGWVQSMLTELNPPSSAFASSSQQTPIDDPLLAPSESST
VvDELLA3    LERLETVMVNAPSE-ISQLASDVLHCNPSDIASWVDSMLFEENP--------------------------

   AtRGA    S------NGLDPVLPSPEICGFPASDYDLKVIPGNAIYQFPAIDSSSS-S----NNQNKRLKSCSSPDSM
VvDELLA1    ISPLDYTNCSTQPKQEPSIFDSPSLDYDLKAIPGKALYSHIEQPPQQPPAPPLYQRDNKRLKPTTSATAN
VvDELLA2    ITTLDFSGSRQQ-SEHQSRIYNDNSDYDLSAIPGVAVYRRPDQQGDG-------EKSSKRMKTASSSSSS
VvDELLA3    ----------------PVSIPSDLSDFSDLTYIDSVVSPAINQTVCN--------------NSWVEHHTP

   AtRGA    VTSTSTGTQIGGVIGTTVTTTTTTTAAGESTRSVILVDSQENGVRLVHALMACAEAIQQNNLTLAEALV
VvDELLA1    SVSSVIG----------------GWGVPTESARPVVLVDSQETGIRLVHTLMACAEAVQQENLKLAEALV
VvDELLA2    STQQVQEN---------------GLASVAESTRPVVVVDSQETGVRLVHTLMACADAVQQDNMKLADALV
VvDELLA3    -----EQQQ-----------------QELTHQLTVVTAMEEDSGIRLVHMMMTCAESVQRGDLPLAGSLI

   AtRGA    KQIGCLAVSQAG--AMRKVATYFAEALARRIYRLSPPQNQIDHCLSDTLQMHFYETCPYLKFAHFTANQA
VvDELLA1    KQIGFLAVSQAG--AMRKVATYFAEGLARRIYRLYP-DKPLDSSFSDILQMHFYETCPYLKFAHFTANQA
VvDELLA2    KHIGLLAASQAG--AMRKVATYFAEALARRIYRIYP-QDSLESSYSDILQMHFYEACPYLKFAHFTANQA
VvDELLA3    EEMQALLTRVNTGCGIGKVARYFIDALNRRVFTPQAPCATGWSNENEILYHHFYEACPYLKFAHFTANQA

   AtRGA    ILEAFEGKKRVHVIDFSMNQGLQWPALMQALALREGGPPTFRLTGIGPPAPDNSDHLHEVGCKLAQLAEA
VvDELLA1    ILEAFEGKKRVHVIDFSMKQGMQWPALMQALALRPGGPPSFRLTGIGPPSTDNTDHLHEVGWKLAQLAET
VvDELLA2    ILEAFAGANRVHVIDFGLKQGMQWPALMQALALRPGGPPSFRLTGIGPPQPDNTDALQQVGWKLAQLAET
VvDELLA3    ILEAFDGHDCVHVVDFNLMHGLQWPALIQALALRPGGPPLLRLTGIGPPSPDGRDSLREIGLRLAELARS

   AtRGA    IHVEFEYRGFVANSLADLDASMLELRPSDTEAVAVNSVFELHKLLG----RPGGIEKVLGVVKQIKPVIF
VvDELLA1    IHVEFEYRGFVANSLADLDASMLELR--DGESVAVNSVFELHSLLA----RPGGIERVLSAVKDMKPDIV
VvDELLA2    IGVEFEFRGFVANSLADLEPSMLQIRPPEVEAVAVNSVLELHRLLA----RPGAIEKVLSSIKAMKPKIV
VvDELLA3    VNVRFAFRGVAASRLEDVKPWMLQVSP--KEAVAINSIMQLHRLLGSGPTRVSAIETVLGWIRSLNPKIV

   AtRGA    TVVEQESNHNGPVFLDRFTESLHYYSTLFDSLEGVPNS----QDKVMSEVYLGKQICNLVACEGPDRVER
VvDELLA1    TIVEQEANHNGPVFLDRFTESLHYYSTLFDSLEGCGVSPVNTQDKLMSEVYLGQQICNVVACEGPERVER
VvDELLA2    TVVEQEASHNGPVFLDRFTEALHYYSNLFDSLEGCGVSPPSSQDLMMSEIYLGRQICNVVACEGAERVER
VvDELLA3    TVVEQEANHNQSEFLDRFTEALYYYSTMFDSLEACSLQP----EKAVAEIYIQKEICNVLCCEGSARVER

   AtRGA    HETLSQWGNRFGSSGLAPAHLGSNAFKQASMLLSVFNSGQGYRVEESNGCLMLGWHTRPLITTSAWKLST
VvDELLA1    HETLAQWRARLGSAGFDPVNLGSNAFKQASMLLALFAGGDGYRVEENNGCLMLGWHTRPLIATSAWQLAN
VvDELLA2    HETLSQWRSRMGSAGFDPVHLGSNAFKQASMLLALFAGGDGYRVEENNGCLMLGWHTRPLIATSAWQLNS
VvDELLA3    HEPLAKWRNRLGAAGFRPLNLGSNAFKQASMLLTLFS-AEGYCVEEHDGCLTLGWHSRPLIAASAWQPLL

   AtRGA    AAY-------------
VvDELLA1    KPALPSSTPASN----
VvDELLA2    NFNPTSTSASTSAHSQ
VvDELLA3    DTVINHNNLL------
```

S1C

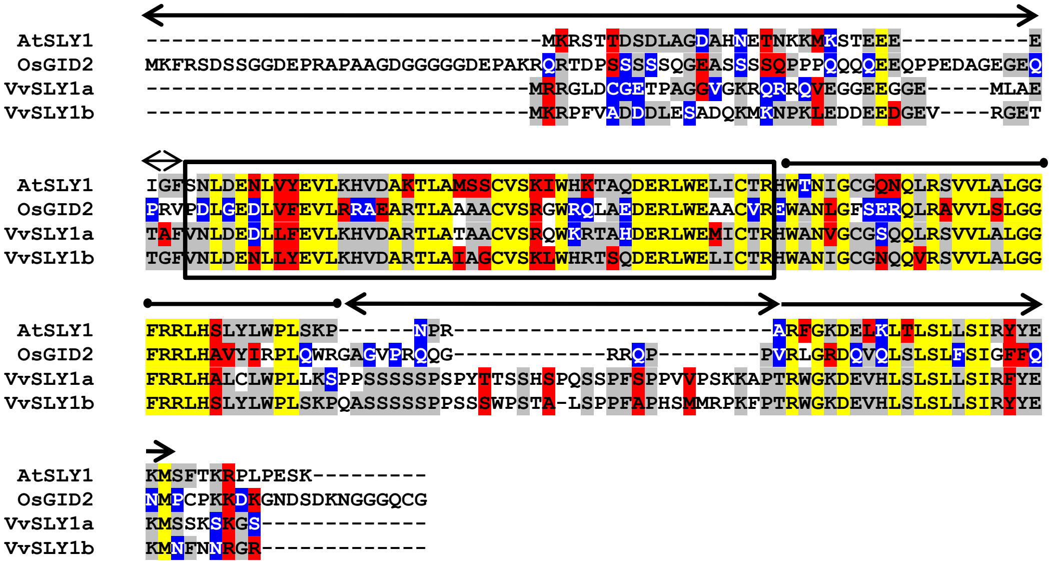


Figure S1: **Amino acid sequence alignment of major GA signaling genes.** Multiple alignment of amino acid sequences of Arabidopsis, rice and *V. vinifera* gene families were generated by CLUSTAL W alignment algorithm using AlignX of the Vector NTI suite. Yellow-shade: identical residues; Red-shade: similar residues; Blue-shade: weakly similar residues; Grey-shade: conservative residues; Unshaded: non-similar residues. For accession numbers of genes, refer to Material and Methods section. **(A)** VvGID1 paralogs (VvGID1a, VvGID1b), and orthologs from Arabidopsis (AtGID1a, AtGID1b, AtGID1c) and rice (OsGID1) share conserved domains characteristic of soluble GA receptor. Boxed: catalytic triad of Hormone Sensitive Lipases (HSL); Solid circles: Conserved residues in GID1s (the first two pair forming oxyanion with the last); Solid squares: residues essential for interacting with bioactive GA; Diamond-shaped: residues essential for GID1-DELLA interactions; Arrows: sequence variation between VvGID1a and VvGID1b in motif required for DELLA interaction. **(B)** Sequence alignment of VvDELLA paralogs [VvDELLA1 (previously characterized as VvGAI1), VvDELLA2, and VvDELLA3], and ortholog from Arabidopsis (RGA). Arrows: putative VvDELLA sequence variations in motifs required for GID1 interaction; Diamond-shaped: variations in residues required for DELLA protein stabilization. **(C)** VvSLY1 paralogs (VvSLY1a and VvSLY1b), and orthologs from Arabidopsis (AtSLY1) and rice (OsGID2). Double-headed arrows: variable regions; Boxed: F-box domain; Circle-edged lines: GGF domain; Single-headed arrows: LSL domain

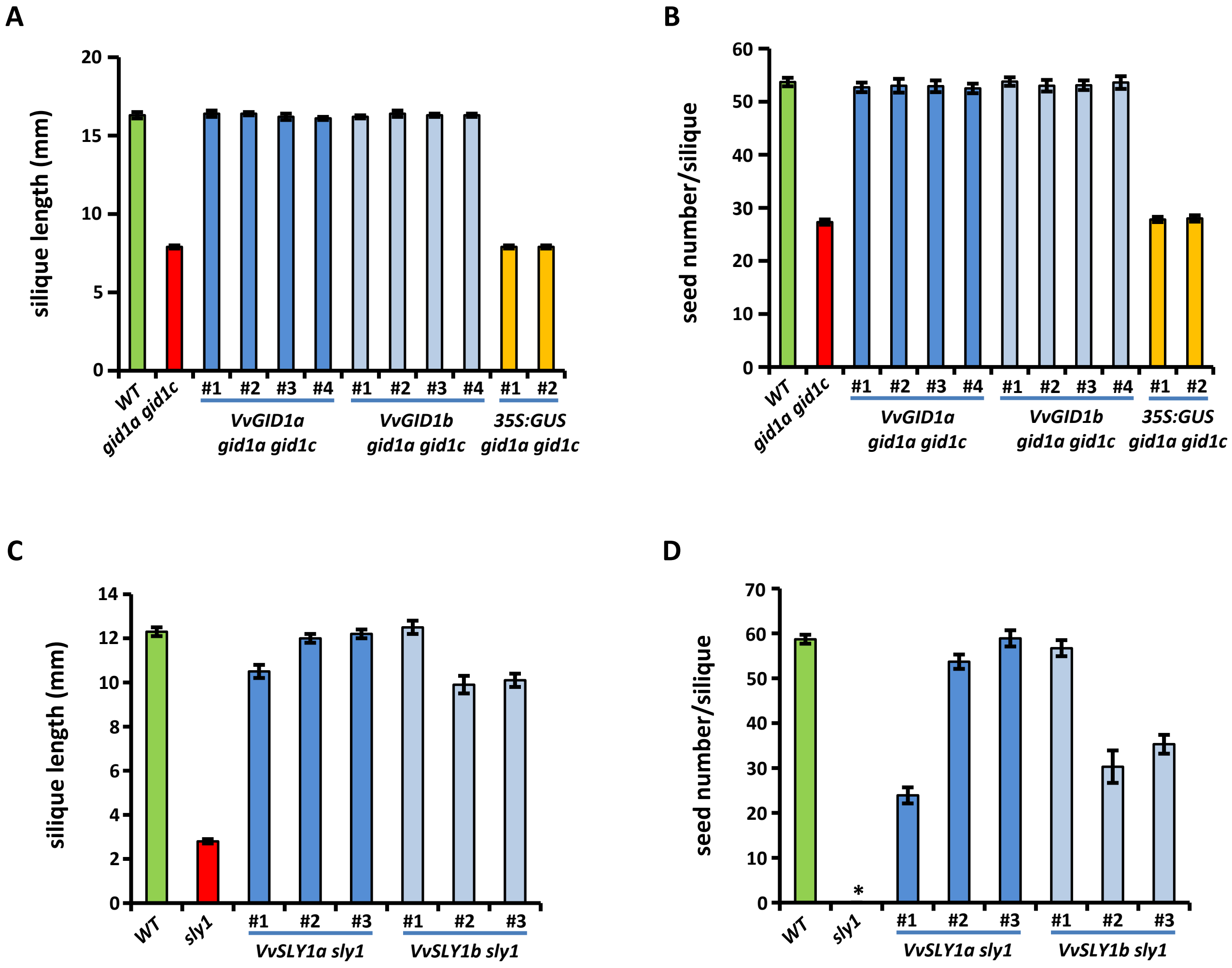


Figure S2 : **Grapevine GA signaling genes rescue silique length and fertility defects of corresponding Arabidopsis mutants. (A-B)** Average silique lengths (A) and seed numbers (B) of WT, *gid1a-2 gid1c-2*, and four representative *35S:VvGID1 gid1a gid1c* transgenic lines. *35S:GUS gid1a gid1c* transformants were included as negative control. **(C-D)** Average silique lengths (C) and seed numbers (D) of WT, *sly1-10*, and three representative *35S:VvSLY1 sly1-10* transgenic lines. In A-D, the length and seed number of 10$^{th}$ silique on the main stem were measured, and both were significantly different from the corresponding mutant (n≥9; $p$<0.01). In contrast, the *35S:GUS gid1a gid1c* lines have no phenotypic difference from *gid1a gid1c* (n≥10). Parameters for VvGID1, VvSLY1 transformants were measured at 51 and 87 days, respectively. Asterisk: no viable seed.

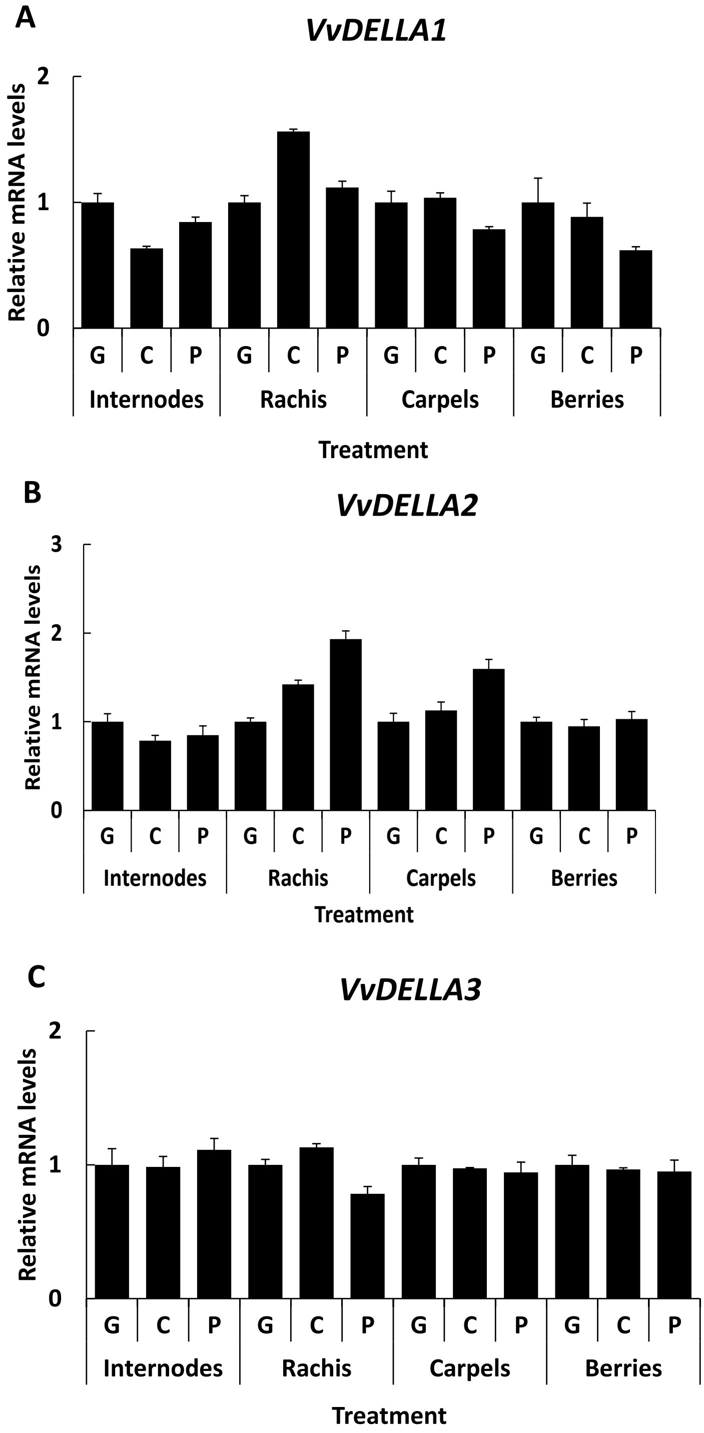


Figure S3: **GA regulation of expression of *VvDELLA1* (A), *VvDELLA2* (B), and *VvDELLA3* (C).** Organs were dipped or sprayed until run-off with a single $GA_3$ (G), paclobutrazol (P), or Triton X-100 (C) treatment. Tissues/organs were sampled 6 h after GA treatment (121 μM for internodes and rachises, and 90 μM for carpels and berries), and 102 h after PAC treatment. Total RNA was extracted from pooled samples of each treatment. The absolute mRNA levels of each gene were determined by real-time quantitative RT-PCR (qRT-PCR) and normalized against *VvGAPDH*. Absolute expressions of gene, in any organs/tissues are shown relative to values of GA-treated. The values represent the mean ± SE of three biological repeats with two technical repeats each. Results were reproducible in successive growing seasons.

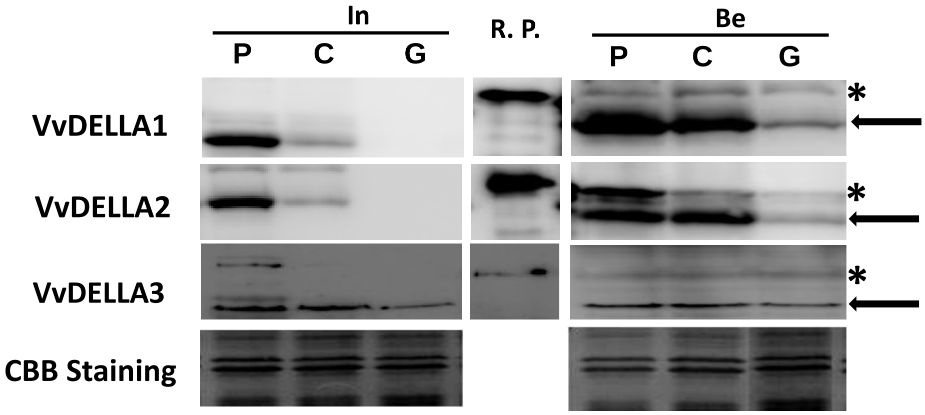


Figure S4: **$GA_3$-induced degradation of VvDELLA proteins in cv. Thompson seedless.** Western blot analyses of VvDELLA proteins in organs using protein-specific, affinity-purified, anti-VvDELLA polyclonal antibodies. Total proteins were extracted from internodes (In) and berries (Be) treated with PAC (P, 0.8 mM) and $GA_3$ (G, 121 μM for internodes, and 90 μM for berries). Control (C) samples were treated with Triton X-100 (0.025%). Recombinant full-length proteins (R.P.) (3.75 ng each of VvDELLA1 and VvDELLA2, and 37.5 ng of VvDELLA3) were used as size controls. Coomassie Brilliant Blue-stained (CBB) proteins were used as loading control. In all lanes except R.P., solid black arrows show band of interest, and Asterisked-bands indicate non-specific proteins detected by the anti-VvDELLA antibodies. Differences in sizes of R.P. and endogenous VvDELLA proteins result from tags on the R.P.

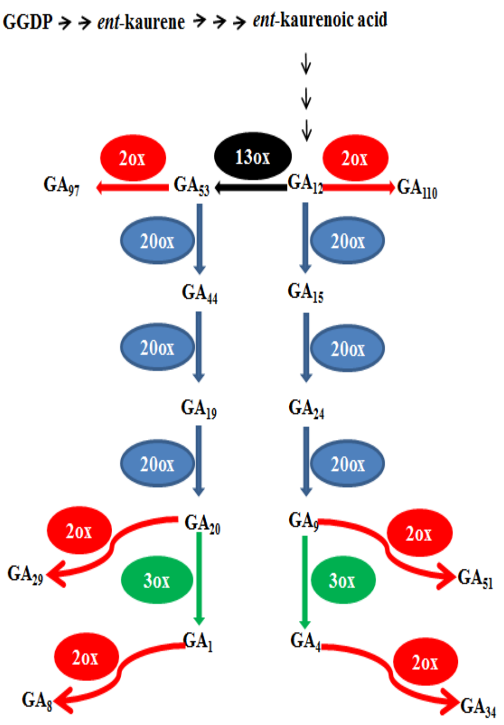


Figure S5: **Schematic representation of major GA metabolism pathways in plants.**
Number of short black arrows indicates number of intermediate molecules in the multiple reactions. Geranylgeranyl diphosphate (GGDP) is converted to *ent*-kaurene and then to *ent*-kaurenoic acid. *ent*-Kaurenoic acid is converted to $GA_{12}$ in stepwise reactions by *ent*-kaurenoic acid oxidases. Using $GA_{12}$ as substrate, GA13ox produces $GA_{53}$ (thick black arrow), the main precursor of bioactive $GA_1$ in the 13-hydroylated pathway. Through a series of reaction catalyzed by two types of 2-ODDs, GA20ox (solid blue arrow) and GA3ox (solid green arrow), $GA_{12}$ and $GA_{53}$ are converted to bioactive $GA_4$ and $GA_1$, respectively. Deactivation of $GA_1$ and $GA_4$ and their precursors proceed through the action of another 2-ODD, GA2ox (solid red arrows).

**Table S1:** Primers used for gene isolation and gene expression analyses by qRT-PCR

| Gene | Sequence | | Purpose | |
|---|---|---|---|---|
| | Forward | Reverse | Gene isolation | qRT-PCR |
| *VvGID1a* | CACCATGGCCGGGAGT | AAGTCTATTAACAGTTAGAACTCAC | √ | |
| *VvGID1b* | CACCATGGCCGGGAGT | TTAACAGTTAGATTTCACGAAATTC | √ | |
| *VvDELLA1* | CACCATGAAGAGGGAGTATCA | ACTCAGTTGGAGGCAGGTGT | √ | |
| *VvDELLA2* | CACCATGAAGAGAGACCTCCT | TCACTGAGAATGAGCAGAGGT | √ | |
| *VvDELLA3* | CACCATGGGGCCTTACGAC | GAGGAGATTATTATGAT | √ | |
| *VvSLY1 a* | CACCATGAGGCGAGGACTGGA | TTAACTGCCTTTGCTTTTGGA | √ | |
| *VvSLY1 b* | CACCATGAAGCGACCTTTTG | TCAACAATAGAGGGAGATGA | √ | |
| *VvGID1a* | AGGCTCTTGTGGCAGCATG | CTCCTTATGGCCGGAAGTCA | | √ |
| *VvGID1b* | AACTGCCTCTTTCGGTCAAG | AGTTGGTTCAGCCAAGTCTCA | | √ |
| *VvDELLA1* | CGACTCCTCGTTCTCCGATATT | GATAGCTTGATTGGCGGTGAAG | | √ |
| *VvDELLA2* | TGGTGGACGTGGCTCAGA | ACAGTCCCAGAGGAGAGATGAGA | | √ |
| *VvDELLA3* | CTTGGAGCAGCAGGGTTCAG | ACCCCTCAGCCGAGAACAG | | √ |
| *VvSLY1 a* | CGTCGTCTCACTCTCCTCAGT | AAGATGAACCTCGTCCTTTCC | | √ |
| *VvSLY1 b* | GATCTGGAAAGCGCAGATCAG | TACAGCAGATTCTCGTCCAGATTC | | √ |
| *VvGAPDH* | TTCTCGTTGAGGGCTATTCCA | CCACAGACTTCATCGGTGACA | | √ |

**Table S2:** Primers used for cloning genes to yeast two-hybrid vectors

| Gene | Sequence | |
| --- | --- | --- |
| | **Forward (VvGene-1)** | **Reverse (VvGene-2)** |
| *VvGID1a* | CGAGGAATTCATGGCCGGGAGTAATGAAGTCAAC | GCAGGGATCCTTAACAGTTAGAACTCACAAAGTTAC |
| *VvGID1b* | CGAGGAATTCATGGCCGGGAGTGATGAAGTCAAC | GCAGGGATCCTTAACAGTTAGATTTCACGAAATTCTTTATC |
| *VvSLY1a* | CTTCGAATTCATGAGGCGAGGACTGGACTGCG | CCTTGGATCCTTAACTGCCTTTGCTTTTGGAACTC |
| *VvSLY1b* | CTTCGAATTCATGAAGCGACCTTTTGTTGCCGAC | CCC GGATCC TCATCTCCCTCTATTGTTGAAAT |
| *VvDELLA1* | GGCAGCATATGAAGAGGGAGTATCATCATC | GCAGGGATCCTCAGTTGGAGGCAGGTGTGG |
| *VvDELLA2* | GCAGCATATGATGAAGAGAGACCTCCTAGACGGTTG | GCAGGGATCCTCACTGAGAATGAGCAGAGGTGGAG |
| *VvDELLA3* | GCAGCATATGATGGGGCCTTACGACTCTGCCATC | GCAGGAATTCTTAGAGGAGATTATTATGATTTATAAC |

**Table S3:** Amount of deuterated GA species used as the Internal Standard (IS) mix during extraction of endogenous gibberellins in different organs of grapes. Cat. 1= Berries (30 d); Cat. 2= Young leaves, Mature leaves, Young tendrils, mature tendrils, carpels, berries (0 d), berries (10 d); Cat. 3= Young rachis, mature rachis; Cat. 4=Young internodes

| **GA species** | **Amount (ng)/ extraction** | | | |
|---|---|---|---|---|
| | **Cat. 1** | **Cat. 2** | **Cat. 3** | **Cat. 4** |
| $GA_1$ | 0.025 | 5 | 0.5 | 5 |
| $GA_4$ | 0.125 | 0.25 | 0.025 | 0.25 |
| $GA_{53}$ | 0.025 | 5 | 0.5 | 5 |
| $GA_{44}$ | 0.025 | 5 | 0.5 | 5 |
| $GA_{19}$ | 0.025 | 5 | 0.5 | 5 |
| $GA_{20}$ | 0.025 | 5 | 0.5 | 5 |
| $GA_{29}$ | 0.025 | 5 | 0.5 | 5 |
| $GA_8$ | 0.025 | 5 | 0.5 | 5 |
| $GA_{12}$ | 0.125 | 0.25 | 0.025 | 0.25 |
| $GA_{15}$ | 0.125 | 0.25 | 0.025 | 0.25 |
| $GA_{24}$ | 0.125 | 0.25 | 0.025 | 0.25 |
| $GA_9$ | 0.125 | 0.25 | 0.025 | 0.25 |
| $GA_{51}$ | 0.125 | 0.25 | 0.025 | 0.25 |
| $GA_{34}$ | 0.125 | 0.25 | 0.025 | 0.25 |

**Table S4:** GA signaling genes of *Vitis vinifera* cv. Thompson seedless

| Gene name | NCBI Locus ID | Chromosome | Size of gene (bp) | mRNA size (bp) | Protein size (kDa) |
|---|---|---|---|---|---|
| *VvGID1a* | AM468374 | 14 | 1,934 | 1,032 | 38.4 |
| *VvGID1b* | AM479851 | 7 | 1,817 | 1,032 | 38.9 |
| *VvDELLA1* | AM459432.1 | 1 | 1,770 | 1,770 | 64.8 |
| *VvDELLA2* | AM470304.2 | 14 | 1,830 | 1,830 | 66.1 |
| *VvDELLA3* | AM484828.1 | 11 | 1,596 | 1,596 | 58.6 |
| *VvSLY1a* | AM445694.2 | 7 | 552 | 552 | 20.2 |
| *VvSLY1b* | AM450967 | 18 | 552 | 552 | 20.9 |
